# Antimicrobial Peptides: An Update on Classifications and Databases

**DOI:** 10.3390/ijms222111691

**Published:** 2021-10-28

**Authors:** Ahmer Bin Hafeez, Xukai Jiang, Phillip J. Bergen, Yan Zhu

**Affiliations:** 1 Centre of Biotechnology and Microbiology, University of Peshawar, Peshawar 25120, Pakistan; ahmerbenhafeez64@gmail.com; 2 Infection and Immunity Program, Department of Microbiology, Biomedicine Discovery Institute, Monash University, Clayton, VIC 3800, Australia; Xukai.Jiang@monash.edu (X.J.); phillip.bergen@monash.edu (P.J.B.); 3 National Glycoengineering Research Center, Shandong University, Qingdao 266237, China

**Keywords:** antimicrobial peptide, database, structure, mode of action, machine learning, HMM, BLAST

## Abstract

Antimicrobial peptides (AMPs) are distributed across all kingdoms of life and are an indispensable component of host defenses. They consist of predominantly short cationic peptides with a wide variety of structures and targets. Given the ever-emerging resistance of various pathogens to existing antimicrobial therapies, AMPs have recently attracted extensive interest as potential therapeutic agents. As the discovery of new AMPs has increased, many databases specializing in AMPs have been developed to collect both fundamental and pharmacological information. In this review, we summarize the sources, structures, modes of action, and classifications of AMPs. Additionally, we examine current AMP databases, compare valuable computational tools used to predict antimicrobial activity and mechanisms of action, and highlight new machine learning approaches that can be employed to improve AMP activity to combat global antimicrobial resistance.

## 1. Introduction

Antibiotic resistance is a global public health problem. Due to rapidly increasing antibiotic resistance over the past decades, our last-line antimicrobials are beginning to fail and a return to a pre-antibiotic era is a distinct possibility [1,2]. In this environment, research into non-conventional anti-infective agents has intensified, with antimicrobial peptides (AMPs) considered potential drug candidates for the treatment of infections caused by otherwise untreatable microorganisms [3,4]. The first AMP, gramicidin, was discovered in 1939 from the soil bacteria *Bacillus brevis* and showed in vitro and in vivo antibacterial activity against many Gram-positive bacteria [5,6]. Subsequently, the number of catalogued AMPs, also known as host-defense peptides (HDPs), has increased enormously. Most AMPs are oligopeptides of 5 to 100 amino acids with a positive net charge (typically +2 to +11) and a significant proportion (typically 50%) of hydrophobic residues [7,8]. In mammals, they are active against a wide variety of microbes including bacteria, fungi, and unicellular protozoa, as well as viruses [9,10,11,12]. The reported mechanisms of action of AMPs are diverse and generally result in the direct killing of the pathogen, although several AMPs may also kill indirectly via modulating host immune responses [13,14]. An important feature that sets AMPs apart from conventional antibiotics is their attack on multiple low-affinity targets such as bacterial membranes, which is thought to mitigate the development of antimicrobial resistance [15]. AMPs are also amenable to mutagenesis and peptide engineering, properties that have already resulted in the production of numerous compounds with enhanced bioactivity and reduced cytotoxicity [16,17]. In this review, we examine the sources, structures, modes of action, and classifications of AMPs, as well as reasons why the translation of AMPs into the clinic has been slow and how this can be improved. We also review some of the regularly curated and maintained AMP databases and highlight important features and tools associated with the prediction, improvement, and activity of AMPs.

## 2. Sources of AMPs

### 2.1. Bacteriophage/Viral AMPs

Bacteriophages (phages) are viruses that infect bacteria. Many phage proteins including endolysins (lysins), virion-associated peptidoglycan hydrolases (VAPGHs), depolymerases, and holins display antibacterial activity [18,19,20]. These phage AMPs are of two types, namely phage-encoded lytic factors and phage-tail complexes [21].

Phage lysins are peptidoglycan-hydrolyzing enzymes that range in size from 25 to 40 kDa [22]. A detailed description of their mode of action is beyond the scope of this review. Briefly, phage lysins weaken the bacterial cell wall by digesting peptidoglycan, creating holes in the cell wall that permit phage progeny to exit the cell. Lysins are currently being considered as an alternative or adjunct to antibiotics given that they possess a number of distinguishing features: (i) targeting the highly conserved bacterial peptidoglycan which typically results in rapid bactericidal activity, (ii) synergy with cell wall-inhibiting antibiotics, (iii) anti-biofilm activity, and (iv) stability (they can be frozen and lyophilized and are heat stable up to ~50 °C) [23,24,25]. Their activities have been investigated both in vitro and in vivo against a number of Gram-positive and -negative bacteria [26,27]. Examples include PK34 and LysAB2 P3, which exhibit activity against *Mycobacterium tuberculosis* and *Acinetobacter baumannii*, respectively [28,29]. Phage lysin PlyV12 exhibits broad bactericidal activity against enterococci and other Gram-positive organisms including *Streptococcus pyogenes*, *Staphylococcus aureus*, and group B *streptococci* [30].

VAPGHs are mostly encoded by double-stranded DNA phages and consist of a *C*-terminal cell wall binding domain and one or more *N*-terminal catalytic domains [31]. They typically result in rapid bactericidal activity and exhibit near species or genus specificity [32,33], thermostability, and functional modularity [34]. Binding of a VAPGH to a bacterial cell occurs via a specific receptor located on the bacterial cell surface and is followed by local hydrolysis of the cell wall, enabling the phage to inject its genetic materials into the bacterial cell [19,35]. VAPGHs can be classified into three categories based on the peptidoglycan cleavage site, namely glycosidases (which cleave one of two glycosidic bonds in peptidoglycan chain), amidases (which cleave amide bonds between *N*-acetylmuramic acid lactyl groups and stem peptide l-alanines), and endopeptidases (which cleave the peptide bonds within either the stem peptide or cross-link) [36]. Most VAPGHs are glycosidases, an example being the lytic transglycosidase Gp16 from phage T7 which cleaves the β-1,4-glycosidic bond between *N*-acetylmuramic acid and *N*-acetyl-D-glucosamine [37,38]. VAPGHs exhibit activity against both Gram-positive and -negative bacteria. For example, bacteriophage vB_SauS-phiIPLA88 contains HydH5 and is active against *S. aureus* [34]. Phage Ф6 contains protein P5 (an endopeptidase), which is active against the Gram-negative bacteria *Pseudomonas aeruginosa*, *Pseudomonas phaseolicola* HB10Y, *Pseudomonas fluorescens*, *Pseudomonas putida*, *Escherichia coli*, *Salmonella enterica* serovar *typhimurium*, and *Proteus vulgaris* [39]. Similarly, protein gp21, coded by *Xanthomonas oryzae* phage Xop411, exhibits killing activity against the *Xantomonas* genus and the Gram-negative species *Stenotrophomonas maltophilia* and *P. aeruginosa* [40].

The phage polysaccharide depolymerases are carbohydrate active enzymes that recognize and degrade polysaccharides of the host bacterial envelope [41]. For example, the genome of *Klebsiella* phage ΦK64-1 encodes multiple depolymerases active against a variety of *Klebsiella* capsular polysaccharides (K1, K11, K21, K25, K30, K35, K64, K69, KN4, and KN5) [42,43], whereas *P. putida* phage AF is capable of degrading the extracellular polysaccharides involved in the *P. putida* biofilm matrix [44]. Because of potential anti-biofilm effects, depolymerases may be effective against biofilm-forming bacteria, including *P. mirabilis*, *E. coli*, *S. suis*, *K. pneumoniae*, and *P. aeruginosa* [45,46,47].

Holins are small sized hydrophobic proteins (<150 amino acids) involved in regulating the time of bacterial lysis via guiding the phage muramidases to the peptidoglycan layer [48,49,50]. They are classified into two different types based on the type of lesion formed, namely canonical holins and pinholins [50,51]. The canonical holins form large pores in the cytoplasmic membrane and allow the secretion of non-specific endolysins and other proteins into the cytoplasm [51,52]. In contrast, pinholins create small holes in the membrane that lead to depolarization prior to peptidoglycan attack [53]. HolGH15 produced by the *S. aureus* bacteriophage GH15 possesses broad antibacterial activity against a variety of pathogens including *S. aureus*, *Listeria monocytogenes*, *Bacillus subtilis*, *Klebsiella pneumonia*, *E. coli*, and *Pseudomonas aeruginosa* [54,55]. HolSD produced by *Streptomyces avermitilis* bacteriophage phiSASD1 exhibit anti-E.coli activity [56].

### 2.2. Bacterial AMPs

#### 2.2.1. AMPs from Gram-Positive Bacteria

**Figure 1:**
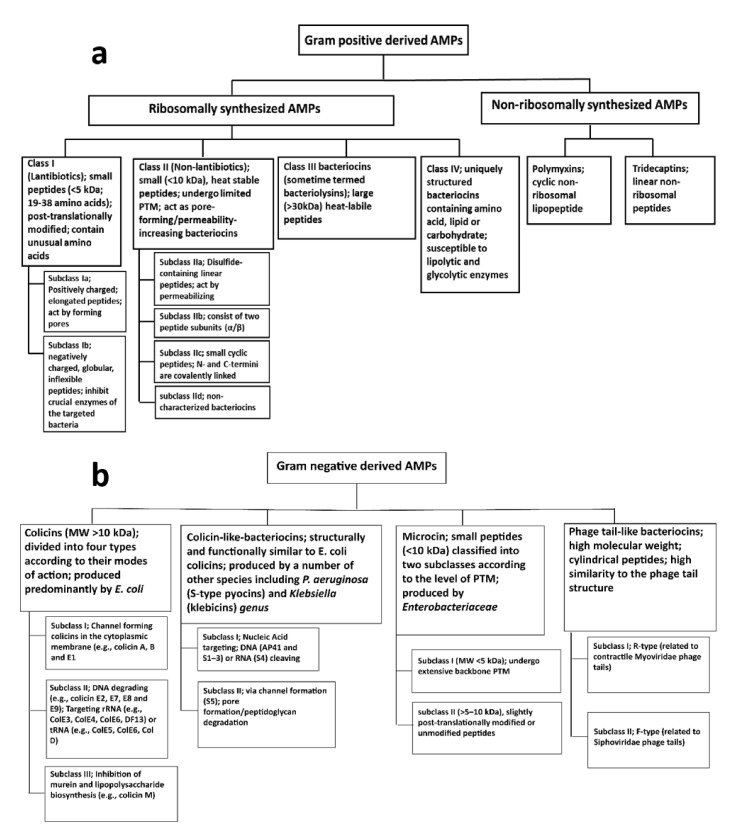
Classification of bacteriocins produced by (**a**) Gram-positive and (**b**) Gram-negative bacteria.

Both ribosomally and non-ribosomally synthesized AMPs have been reported in Gram-positive bacteria (Figure 1a) [57,58]. Ribosomally synthesized bacterial AMPs are termed bacteriocins [59]. These peptides are only active against bacteria closely related to the producing strain, with the producer having a degree of immunity [60]. Bacteriocins produced by Gram-positive bacteria can be classified into four classes: (I) lantibiotics, (II) non-lantiboitics, (III) large-sized bacteriocins, and (IV) uniquely structured bacteriocins [61].

Class I (lantibiotics) contains small peptides (<5 kDa; 19–38 amino acids) which are stable to heat, a wide range of pH levels and proteolysis [62,63], with activity against primarily Gram-positive bacteria [61,64]. Unusual amino acids such as lanthionine and β-methyllanthionine are incorporated into lantibiotics via post-translational modifications (PTMs; e.g., dehydration, thioethers formation, lysinoalanine bridges, oxidative decarboxylation) to enhance structural stability [65,66,67]. Lantibiotics are further divided into subclasses Ia and Ib. Subclass Ia includes nisin (the first and most prominent lantibiotic), epidermin, gallidermin, and Pep5 [68]. This subclass consists of positively charged elongated peptides and usually acts by forming pores in bacterial membranes which leads to an efflux of small molecules, dissipation of membrane potential, and, ultimately, arrest of cellular biosynthesis [68]. However, nisin and epidermin have a dual mode of action that also includes interference with cell wall synthesis via binding to lipid II (a precursor of peptidoglycan), thereby inhibiting the transgylcosylation step in peptidoglycan polymerization [69,70,71]. In addition, binding to lipid II also enhances pore formation [72]. Subclass Ib consists of negatively charged, globular, and inflexible peptides that inhibit crucial enzymes of the targeted bacteria and includes lacticin 481, cytolysin, and salivaricins [61]. The salivaricins act by first binding to lipid II which is followed by pore formation in the cytoplasmic membrane or interference with cell wall synthesis, ultimately leading to cell death [73,74,75]. Cytolysin targets membranes and forms pores, causing osmotic lysis of the cell [76,77].

Unlike the Class I lantibiotics, Class II AMPs are non-lanthionine-containing bacteriocins that undergo limited PTM (restricted to bisulfide bridge formation in a few members such as pediocin AcH and PA-1) and therefore do not contain unusual amino acids [78,79]. They are small (<10 kDa), heat stable peptides that act as pore-forming/membrane-destabilizing/permeability-increasing bacteriocins [80,81]. Class II bacteriocins can be further classified into four subclasses [82]. Subclass IIa consists of disulfide-containing linear peptides with similar amino acid sequences that exhibit strong anti-listerial activity (e.g., leucocin A, acidocin A, pediocin PA-1, and enterocin P) [83,84,85]. All members of this class act by permeabilizing the cell membrane. For example, pediocin PA-1 (the same as pediocin AcH) from *Lactobacillus plantarum* or *Pediococcus* sp. acts by pore formation resulting in dissipation of the proton motive force [86,87]. Subclass IIb bacteriocins consist of two peptide subunits (α/β) in equal proportion which function as a single unit; both are necessary for antimicrobial activity [79,88]. Examples include plantaricin EF and JK, NC8, thermophilin 13, lactacin F, and lactococcin G and Q [79]. Subclass IIb bacteriocins act by increasing the permeability of the target bacterial cell membrane to specific small molecules [79]. For example, lacticin F increases permeability specifically to K^+^ and phosphate [89], whereas lactococcin G increases permeability to a variety of monovalent cations (excluding H^+^) but not divalent cations or anions [90,91]. Subclass IIc is composed of small cyclic peptides whose *N*- and *C*-termini are covalently linked [86]. They include enterocin AS-48, gassericin A, acidocin B, circularin A, lactocyclicin Q, and uberolysin [64,92,93]. The mechanism of action of sublass IIc peptides is similar to that of most bacteriocins, namely permeabilization of the membrane, ion leakage leading to dissipation of the membrane potential, and, ultimately, cell death [94]. All remaining non-characterized bacteriocins in the Class II group share no significant sequence similarity with the other class II bacteriocins and are assigned to subclass IId [61]. Examples include lactococcin A, B, and 972, enterocin L50, and lacticin Q [86].

Class III bacteriocins (sometime termed bacteriolysins [82]) consist of large (>30 kDa), heat-labile peptides [61]. Examples include zoocin A, lysostaphin, enterolysin A, and heleveticin M, J, and V, which have endopeptidase-like activity against peptidoglycan and cause cell wall disruption [95,96,97]. Class IV AMPs are uniquely structured bacteriocins containing amino acid and lipid or carbohydrate components that make them susceptible to many lipolytic and glycolytic enzymes [61,98]. Examples include plantaricin S and leuconocin S, lactocin 27, and pediocin SJ-1, which have cell membrane-disrupting activity [99,100].

Polymyxins are a class of cyclic non-ribosomal lipopeptide antibiotics isolated from the Gram-positive spore-forming soil bacterium *Paenibacillus polymyxa* (previously known as *Bacillus polymyxa*) [101,102]. They consist of a cyclic heptapeptide with a tripeptide side chain linked to an *N*-terminal fatty acyl tail [103]. Thus far, 10 different groups of polymyxin lipopeptides have been identified (classified as polymyxins A, B, C, D, E, F, M, P, S, and T), although only polymyxin B and E (the latter known as colistin) are used clinically [103]. Both polymyxin B and E are active against a range of important Gram-negative bacteria including *Enterobacterales* as well as *A. baumannii*, *P. aeruginosa*, and *S. maltophilia* [103]. Importantly, they retain activity against Gram-negative bacteria listed as Priority 1 (Critical) pathogens by the World Health Organization (WHO) [104]. The known mechanism of action includes disruption of the Gram-negative outer membrane, inhibition of vital respiratory enzymes (type II NADH-quinone oxidoreductases) in the bacterial inner membrane [105], and induction of a hydroxyl radical death pathway [106].

#### 2.2.2. AMPs from Gram-Negative Bacteria

To date, the majority of bacteriocins isolated from Gram-negative bacteria have been reported in *E. coli*, although other species including *Klebsiella* spp. and *Pseudomonas* spp. also synthesize AMPs [107]. These chemicals have a narrow spectrum of activity against Gram-negative organisms and can be classified into four classes: colicins, colicin-like, microcins, and phage tail-like bacteriocins (Figure 1b) [61].

The colicins (MW > 10 kDa) are produced predominantly by *E. coli* [108]. Colicins bind to specific cell surface receptors prior to translocation through the outer membrane, periplasm, and inner membrane into the cell cytoplasm [109]; the complicated translocation process has been reviewed by Cascales et al. (2007) and is beyond the scope of this review. Examples of colicin binding include colicin A, K, and U which bind to BtuB, Tsx, and OmpA receptors, respectively [108]. The production of colicins kills not only sensitive neighbouring cells but also the producing cells [109]. Colicins can be divided into four types according to their modes of action: (i) channel formation in the cytoplasmic membrane (e.g., colicin A, B, and E1), (ii) DNA degradation (e.g., colicin E2, E7, E8, and E9), (iii) targeting of rRNA (e.g., ColE3, ColE4, ColE6, and DF13) or tRNA (e.g., ColE5, ColE6, and Col D), and (iv) inhibition of murein and lipopolysaccharide biosynthesis (e.g., colicin M) [109,110]. Colicin-like-bacteriocins are structurally and functionally similar to *E. coli* colicins but are produced by a number of other species including *P. aeruginosa* (S-type pyocins) and *Klebsiella genus* (klebicins) [111,112]. S-type pyocins (AP41 and S1–S5) are sensitive to proteases and induce cell death by cleaving DNA (AP41 and S1–S3) or RNA (S4), or via channel formation (S5) [113]. Klebicins act via endonuclease activity, pore formation, and/or peptidoglycan degradation [107,108,112].

Microcins are produced by *Enterobacteriaceae* and are active against phylogenetically close species [114,115]. They consist of small peptides (<10 kDa) classified into two subclasses according to the level of PTM [116]. Subclass I contains microcin B17, C7, D93, and J25 (MW < 5 kDa), which have undergone extensive backbone PTM, whereas subclass II includes larger (5–10 kDa), slightly post-translationally modified or unmodified peptides [117]. Examples of subclass II include microcin E492, V, L, and H47. Many modes of action for the microcins have been described, reflecting their diverse cellular targets. For example, E492 targets the bacterial membrane via pore formation and membrane potential disruption [118], whereas B17 interferes with replication by targeting the DNA gyrase [119]. J25 likely has a dual mechanism of action involving interference with RNA-polymerase [120] and membrane disorganization [121].

Phage tail-like bacteriocins are high molecular weight cylindrical peptides, so named due to their high similarity to the phage tail structure [122,123]. Phage tail-like bacteriocins can be divided into two major class, namely the R-type (related to contractile *Myoviridae* phage tails) and F-type (related to *Siphoviridae* phage tails) [122]. The most studied phage tail-like bacteriocins are the R- and F-pyocins from *P. aeruginosa* [113]. The R-type phage tail-like bacteriocins initially bind to cell surface receptors after which the sheath (a polymer of a single polypeptide) contracts, forcing the internal core into the cell envelope. This process leaves a channel in the cell envelope through which ions flow, decoupling cellular ions gradients and respiration and resulting in rapid cell death [122]. The R-type phage tail-like baceriocins also interfere with oxygen uptake and macromolecule synthesis [124]. While the F-type phage tail-like bacteriocins lack a contractile mechanism, it is thought that a similar channel to the R-types nonetheless forms in the inner membrane, resulting in killing via a similar mechanism [125].

### 2.3. Fungal AMPs

Fungal AMPs can be divided into peptaibols and fungal defensins [126,127]. The peptaibols are mainly derived from the soil fungi *Trichoderma* [128]. They are short peptides of 5–21 amino acids, contain a high proportion of non-proteinogenic amino acids such as α-aminoisobutyric acid (Aib), and typically have an acylated *N*-terminal residue and an amino alcohol (e.g., phenylalaninol or leucenol) attached to the *C*-terminal [129]. Their name derives from three of their characteristic components: peptide, Aib, and amino alcohol [130]. The peptaibol database contains 317 molecules (http://peptaibol.cryst.bbk.ac.uk/home.shtml (accessed on 2 October 2020)) [131]. The most widely studied peptaibol is alamethicin, which was isolated from *T. viridea* and is active against both Gram-positive (*E. faecalis*, *S. hemolyticus*, *S. aureus*, and *Streptococcus viridans*) and -negative (*E. coli*, *K. pneumoniae*, *P. vulgaris*, and *P. aeruginosa*) bacteria [132] and fungi [129]. Other peptaibols include trichogin GA IV from *T. longibrachiatum*, a 10-amino acid, protease-resistant peptide with an *N*-terminal acyl chain, Aib residues, and a C-terminal leucinol [133]; tricholongin B (BI and BII), a group of 19-amino acid, highly hydrophobic peptides from *T. longibrachiatum* which are active against fungi and Gram-positive bacteria [134]; saturnisporin SA (SAII and SAIV), from *T. Saturnisporum*, a 20-amino acid peptide active against *S. aureus* [135]; sillucin, from *Rhizomucor pusillis*, a 30-amino acid AMP containing four disulfide bridges with activity against Gram-positive bacteria [136,137]. The primary mechanism of action of all peptaibols is similar and primarily involves membrane disruption [138]. Based on chain length, peptaibols are classified into short-chain (5-10 amino acids), medium-chain (11–16 amino acids), and long-chain (17–21 amino acids) peptaibols [138]. Classification based on sequence similarity has also been suggested [139]. Larger peptaibols (≥15 amino acids) can form helical structures that oligomerize and form ion channels in the membrane [139,140]. The action of shorter peptaibols (<15 amino acids) is more complex, with activity likely resulting from a combination of membrane disruption (e.g., formation of transmembrane channels via helical bundles within the bilayer or by a barrel stave mechanism) and effects on different molecular targets [141,142].

Defensins are short, cysteine-rich peptides that are widely distributed across microorganisms, plants, and animals [143]. The fungal defensins are named defensin-like peptides (DLPs) due to their high sequence and structural similarities [144]. Plectasin, from *Pseudoplectania nigrella*, was the first characterized fungal defensin and exhibits activity against predominantly Gram-positive bacteria including *S. pyogenes*, *C. jeikeium*, *C. diphtheriae*, and *S. aureus* [145,146]. Plectasin is structurally similar to plant and insect defensins and contains a core structural motif of a cysteine-stabilized α/β-fold [146]. Whereas many defensins are thought to act via disruption of the microbial cytoplasmic membrane, plectasin acts by binding directly to the bacterial cell-wall precursor lipid II, thereby inhibiting cell wall biosynthesis [145]. Copsin (produced by *Coprinopsis cinerea*) acts similarly to plectasin and is active against a variety of Gram-positive bacteria including *L. monocytogenes*, *M. luteus*, *B. subtilis*, and *E. faecium* [147]. Micasin (from *Microsporum canis*) has broad-spectrum antibacterial activity including against *P. aeruginosa* and methicillin-resistant *S. aureus*; it possibly acts by affecting protein folding, although further studies are required [148].

### 2.4. Plant Derived AMPs

Cysteine-rich AMPs form part of the plant defense systems. They contain multiple disulfide bridges (2 to 6) that result in a compact conformation and confer stability against chemical, thermal, and proteolytic degradation [149]. Plant AMPs are classified into various families according to their cysteine motifs, sequence similarity, and arrangement of disulfide bridges. These families (described briefly in Table 1) include α-hairpinin, defensins, hevein-like peptides, knottin-type peptides (linear and cycle), lipid transfer proteins, thionins, snakins, and unclassified cysteine-rich AMPs [149,150].

### 2.5. Animal Derived AMPs

#### 2.5.1. Invertebrates

Invertebrates lack an adaptive immune response and synthesize AMPs as an integral component of humoral defense [166,167]. Examples of invertebrate AMPs include those found in insects (defensins and cecropins), molluscs and nematodes (defensins), horseshoe crabs (big defensins), invertebrate β-defensins, and crustaceans (crustins) [168]. They are generally classified based on either ancestral studies or sequence analysis. When based on ancestral studies, defensins are classified into two superfamilies: (i) *Cis*-defensins, a large family distributed across the fungal, plant, and animal kingdoms, and (ii) *Trans*-defensins, a small family consisting of invertebrate big defensins and vertebrate defensins [169]. *Trans*-defensins are further classified into α-defensins, β-defensins, θ-defensins (related to α-defensins), and big defensins based on the arrangement of disulfide bridges between six conserved cysteine residues and their overall 3D structure [169,170]. When sequence analysis of conserved cysteine patterns is used for classification, invertebrate AMPs can be classified into five groups: (I) arthropod and mollusc-type 6-cysteine defensins (arthropod defensins), (II) mollusc-type 8-cysteine defensins, (III) nematode-type 8-cysteine defensins, (IV) invertebrate big defensins, and (V) invertebrate (putative) β-defensin-like peptides [171,172].

Defensins, the major AMPs in invertebrates, are cationic peptides that contain six or eight cysteine residues which form three or four disulfide bridges and constitute the common cysteine-stabilized α/β motif [172]. Invertebrate defensins are generally classified into two types according to the number of cysteine residues. The largest group, from arthropods, insects, and mollusks, contains six cysteine residues whereas those in nematodes and mollusks contain eight cysteine residues [171]. They are synthesized as prepropeptides that undergo several proteolytic processing events prior to release as active peptides [173]. Despite sharing no structural or sequence homology to vertebrate defensins, they exhibit a similar mechanism of action to their vertebrate counterparts which involves permeabilizing the cytoplasmic or inner mitochondrial membrane of the target bacteria [174,175,176]. For example, defensin A and sapecin cause cytoplasmic membrane permeabilization via the formation of voltage-dependent channels and by assuming an oligomeric structure in the membrane, respectively [174,175,177,178].

The invertebrate defensins are structurally and phylogenetically related to the vertebrate β-defensins. ‘Big defensin’, initially isolated from the Horseshoe crab (*Trachypleus tridentatus*), showed anti-fungal and anti-bacterial activity [179,180]. Homologous big defensins have subsequently been identified in bivalve mollusks (Bivalvia) and amphioxus (Cephalochordata) [181,182,183]. Big defensin contains two structural domains: a hydrophobic rich *N*-terminal domain with activity against Gram-positive bacteria, and a cationic *C*-terminal domain containing six cysteine residues with activity against Gram-negative bacteria [179]. β-defensin-like peptides have also been found in lobster *Panulirus japanicus* [184] and *P. argus* [185], and are likely involved in important biological activities given their sequence and structural similarity to vertebrate defensins. Cecropins are basic peptides (MW, 4 kDa) synthesized as precursor prepropeptides of 58–64 residues that undergo PTM [186]. Once a threshold concentration has been reached, mature cecropin molecules aggregate on bacterial lipid bilayer membranes causing membrane disruption and subsequent bacterial cell death via a carpet-like model (discussed further in Section 4.2) [187,188,189,190]. They are subdivided into six classes, namely A, B, C, D, and E [191,192]. The primary cecropins are classes A, B, and D and are cationic linear AMPs [193,194]. Classes C, E, and F are present in low amounts and are classified as the degradative products of A, B, and D, respectively (Hultmark et al., 1982). Cecropins are more active against Gram-negative than Gram-positive bacteria [190]. For example, cecropin B possesses 40-fold greater activity against *E. coli* than *S. aureus* [195].

*Crustins* are cationic cysteine-rich peptides found in crustaceans that form a tightly packed structure [196]. The first crustin identified, carcinin, was isolated from the shore crab *Carcinus maenas* and had activity only against Gram-positive bacteria [197]. Subsequent crustins have been indentified in shrimp, crayfish, lobster, and other brachyuran crabs [198]. They are characterized by an *N*-terminal multi-domain rich in glycine, proline or cysteine, and a *C*-terminal whey acidic protein (WAP) domain with four *C*-terminal disulfide bridges [199]. Crustins can be classified into three types (I–III) based on differences in domain organization between the WAP domain and signal sequence, the latter being a putative sequence of 16–24 amino acids at the *N*-terminus that is removed to convert crustins into the active form [198]. Type I crustins are mainly found in lobster, crab, and crayfish [200,201,202] and contain a cysteine-rich region of variable length between the signal peptide sequence and WAP domain. They are only active against Gram-positive bacteria [197]. Type II crustins contain a signal peptide sequence followed by long glycine-rich and cysteine-rich domains (four cysteine residues) at the *N*-terminal, and a cysteine-rich WAP domain (eight cysteine residues) at the C-terminal [198]. They can be further classified into type IIa (active against Gram-positive bacteria [203]) and type IIb (active against both Gram-positive and Gram-negative bacteria [204]). Type III crustins lack glycine- and cysteine-rich regions but contain a proline–arginine-rich domain between the signal sequence and WAP domain. Type IV crustins consists of two WAP domains and are known as double WAP domain (DWD) crustins [199]. Type V crustins were originally discovered in ants and contain an aromatic amino acid-rich region between the WAP and cysteine-rich domains [205].

#### 2.5.2. Fish and Amphibian AMPs

Vertebrates AMPs range in size from 15–200 residues and play an important role in the immediate defense response to microorganisms [206,207] in fish, amphibians, reptiles, birds, and mamalians.

Fish AMPs include cathelicidins, β-defensins, hepicidins, piscidins, and histone-derived peptides [208]. Cathelicidins are cationic AMPs found in secretory granules of immune cells and are activated upon cleavage by elastase and other proteases [209]. The first cathelicidins were isolated from intestinal tissues of *Myxine glutinosa* (Atlantic hagfish) and were designated hagfish intestinal AMP (HFIAP)-1, -2, and -3 [210]. They act by permeabilizing lipid membranes and possess broad-spectrum antimicrobial activity against a number of Gram-negative and -positive bacteria [211]. A glycine-rich cathelicidin from Atlantic Cod (CodCATH) has activity against Gram-negative bacteria [212], whereas two cathelicidins isolated from Rainbow Trout, rtCATH1 (R146-P181) and rtCATH2 (R143-I178), are potentially active against *Lactococcus garvieae* and other Gram-negative fish pathogens [213]. Another rtCATH1 fragment (R151-V186) is active against *L. garvieae* and *Vibrio anguillarum* [214].

Fish defensins are classified as β-defensin-like proteins given that they contain six conserved cysteine motifs [215,216,217,218,219,220]. They were initially identified in zebrafish, Fugu, and tetraodon using a database mining approach [216] and are active against bacteria [208] and fish-specific viruses [218,221,222]. For example, cod β-defensin (*defb*, identified in Atlantic cod, *Gadus morhua*) is active against the Gram-positive bacteria *Planococcus citreus* and *Micrococcus luteus* [223], whereas EcDefensin (from the orange-spotted grouper, *Epinephelus coioides*) inhibits the replication of to two marine fish viruses (SGIV [a DNA virus] and VNNV [an RNA virus]) [221].

Fish hepcidins are cysteine-rich, iron regulating antimicrobial hormones that share a β-sheet-composed hairpin structure linked via four disulfide bonds [224] similar to human hepcidin [225]. In fish, hepcidin are classified as HAMP1 and HAMP2 [208]. HAMP1 is found in both actinopterygian and non-actinopterygian fish; however, HAMP2 has only been reported in actinopterygian fish [226,227,228]. *CsHepcidin* produced by *Cynoglossus semilaevis* is active against *V. anguillarum* and *Edwardsiella tarda* [229], whereas Om-hep1 from *Oryzias melastigmus* is active against Gram-positive (*C. glutamicum* and *S. aureus*) and some Gram-negative (*E. coli* MC1061, *A. hydrophila*, and *Pseudomonas stutzeri*) bacteria, but not the genus *Vibrio* [230].

Piscidins are linear AMPs with an amphipathic, α-helical structure similar to magainins and cecropins [231]. They are classified into seven types (piscidins 1–7) based on their amino acid sequence, length, and biological activity [232]. The piscidin pleurocidin is a highly basic cationic amphipathic peptide with an α-helical structure. It was first isolated from winter flounder (*Pleuronectes americanus*) and is active against a wide variety of Gram-positive and -negative bacteria [232,233].

Amphibians are the largest source of AMPs, with the Antimicrobial Peptide Database (APD, https://aps.unmc.edu/ (accessed on 2 October 2020)) [234] containing 1117 entries from frogs and toads. Amphibian AMPs have been well reviewed [235] and include bombinins, buforin, cathelicidin, dermaseptins, esculentins, fallaxin, magainins, maximins, phylloseptins, phylloxin, plasticins, plasturins, pseudins, and ranateurins (Table 2).

#### 2.5.3. Reptile- and Avian-Derived Peptides

Reptile and avian AMPs are members of the cathelicidin and defensin families [266,267]. Cathelicidins are small-sized AMPs secreted from macrophages and neutrophils upon their activation. Examples of reptile cathelicidins include OH-CATH, a peptide from king cobra contaning 34 amino acids and active against *E. cloacae*, *Enterobacter aerogenes*, and *P. aeruginosa* [268]; cathelicidin BF, a 30-amino acid peptide produced by banded krait and active against *S. aureus*, *B. cereus*, *Salmonella enterica* Serovar Typhimurium, *E. coli*, and *P. aeruginosa* [269]; omwaprin, a 50-amino acid peptide from the venom of inland taipan (*Oxyuranus microlepidotus*) and active against *Bacillus megaterium* and *Staphylococcus warneri* [270]. We refer readers interested in reptile AMPs to the review by van Hoek [266]. Avian AMPs were first isolated from chicken (Chicken Heterophil Peptides, CHP1 and 2) and turkey (Turkey Heterophil Peptides, THP 1, 2, and 3) [271]. Cathelicidins from chicken are named fowlicidins and are grouped into three types (fowlicidin-1 to -3) [272]; they are active against Gram-positive *S. epidermidis*, *B. subtilis*, and *S. aureus*, and Gram-negative *E. coli*, *P. aeruginosa*, and *S. enterica* [272,273,274]. In chickens, chCATH B1, isolated from a specialized organ for hematopoiesis and B cell development (the avian bursa of Fabricius), is a potential antimicrobial against *S. aureus*, *P. aeruginosa*, and *E. coli* [275]. Cathelicidin-like-peptides have also been found in common quail (cathelicidin Cc-CATH1, 2, and 3 from *Coturnix coturnix*) [276], Japanese quail (Cj-CATH-1, -2, -3, and -B1 from *Coturnix japonica*) [277], rock pigeon (Cl-CATH2 and 3 from *Columba livia*) [278], duck (dCATH from *Anas platyrhynchos*) [279], pheasant (Pc-CATH1, 2, and 3 from *Phasianus colchicus*) [280], and turkey (CATH2 and 3 from *Meleagris gallopavo*) [281,282].

The first β-defensin discovered in reptiles was a 40-residue peptide isolated from leukocytes of the European pond turtle (*Emys orbicularis*) and named turtle β-defensin 1 (TBD-1) [283]. TBD-1 is more active against *L. monocytogenes* and *E. coli* than MRSA and Candida [283]. Other reptilian AMPs include crotamine [284], pelovaterin [285], and turtle egg-white protein (TEWP) [286]. Avian β-defensins include AvBD1-14 from chicken [287], ostricacins from ostrich (e.g., OSP-1 to OSP-4) [288], and mallard duck β-defensins (AvBD2 and AvBD9) [287]. They are grouped according to their sources into heterophil (the predominate granulated leukocyte in the acute inflammatory response) and non-heterophil categories [289]. Heterophil β-defensins are further divided into two subclasses based on the number of homologous residues. The first subclass shares 22 amino acids and includes THP-1, CHP-1, CHP-2, Gal-1, and Gal-1a, whereas the second subclass shares 17 residues and includes THP-2, Gal-2, and Osp-1 [289]. Non-heterophil β-defensins include Gal-3, Gallopavin-1 (GPV-1), and sphenicins (Sphe-1 and Sphe-2) [290,291].

#### 2.5.4. Mammalian-Derived AMPs

The major mammalian AMPs are members of the cathelicidin and defensins families, although AMPs not belonging to these two families include platelet antimicrobial proteins, hepcidins, and dermcidin [292].

While there is great variety in mature cathelicidin sequences, all mature mammalian cathelicidin peptides are cationic with an amphipathic structure that assumes α-helical, β-hairpin, or elongated conformations [293,294]. LL-37, the most well-studied cathelicidin and the only cathelicidin in humans, has an amphipathic structure which is disordered in aqueous solution but forms an α-helix upon membrane interaction [295]. It is activie against a variety of Gram-positive and Gram-negative pathogens and promotes wound healing when applied topically [295,296,297,298]. The antibacterial activity of LL-37 is due to either pore-formation or interference with cell wall formation [299,300,301,302], whereas its anti-biofilm activity results from a reduction in bacterial cell attachment, the stimulation of twitch motility, and the suppression of biofilm-development genes [298].

Cathelicidin 4 (indolicidin) is a tryptophan- and proline-rich, 13-residue peptide from bovine neutrophils with activity against both Gram-positive and -negative pathogens [303]. It acts via membrane pore formation and inhibition of DNA synthesis [304]. Seven varieties of cathelicidin 4 (buCATH4 A-G) have been described in water buffalo, the most potent being buCATH4C which is active against *B. cereus* and *S. aureus* [303].

Protegrins (PG) are cathelicidins from porcine white blood cells (WBCs) that are arginine and cysteine-rich cationic AMPs of 16–18 amino acids with a β-hairpin structure containing two disulfide bonds [305]. The protegrin family consists of five members (PG1–5) [306,307]. PG1, the most thoroughly investigated protegrin, is active against *E. coli*, *P. aeruginosa*, *E. faecalis*, and *S. aureus* (MRSA) [308], while PG4 is active against *B. subtilis* [309]. Bactenecins are AMPs rich in arginine that have been isolated from bovine, ovine, and caprine neutrophilic granules [310]. Their activity is primarily directed against Gram-negative bacteria and they are cytotoxic for rat embryonic neurons, fetal rat astrocytes, and human glioblastoma cells [311]. Bacterial killing results from membrane permeabilization and blockage of RNA synthesis [312]. Three equine cathelicidins (eCATHs) have been described, namely eCATH1–3 [313]; eCATH1 has the highest antimicrobial potency and broadest spectrum of activity, while eCATH-2 shows a more restricted spectrum of activity.

Vertebrate defensins are classified into three sub-families: α, β, and θ [314]. All are synthesized as ‘prepropeptides’, with the mature peptides sharing several common features including a cationic net charge (+1 to +11), short polypeptide sequences (18–45 amino acids), three intramolecular disulfide bonds, and no glycosyl or acyl side-chain modification [314]; their tertiary structures contain turn-like β-strands. The α-defensins are cationic AMPs of 29–35 amino acids with a secondary structure characterized by three anti-parallel β-sheets. They are synthesized by promyelocytes, neutrophil precursor cells, and intestinal Paneth cells [315]. Two α-defensins from guinea pig neutrophils, GNCP1 and GNCP2 (each with 31 residues and 3 intramolecular disulfide bonds), are active against *S. aureus* and *E. coli* [316]. Several rabbit α-defensins have been identified, including NP-1 and NP-2, the two most cationic peptides with a broad spectrum of activity against both Gram-positive and -negative bacteria [317]. Human α-defensins HNP1–4 are secreted by neutrophils [318,319], whereas HD-5 and HD-6 are secreted by Paneth cells located in the intestinal epithelium [320,321,322]. HNP1 is the most well-studied α-defensin with activity against *S. aureus*, *B. subtilis*, *S. epidermis*, and *E. coli* [323] resulting from the inhibition of DNA and protein synthesis [324]. β-defensins are structurally similar to α-defensins but differ in cysteine residue and disulfide bond distribution [325]. The first mammalian epithelia-derived β-defensin, tracheal antimicrobial peptide, was reported from bovine mucosal epithelial cells and showed activity against Gram positive (*S. aureus*) and Gram-negative (*P. aeruginosa*, *K. pneumoniae*, and *E. coli*) pathogens [326]. Lingual antimicrobial peptide was derived from bovine tongue and has activity against *Nocardia farcinia*, *E. coli*, *P. aeruginosa*, *S. aureus*, *C. albicans*, and *C. tropicalis* [327,328]. A 13-membered family (BNBD-1 to -13) of β-defensins was isolated from bovine neutrophils [329]. Four β-defensins (HBD-1 to -4) have been identified in humans [325,330] and are expressed in a variety of cells and tissues including in the epithelial lining of the respiratory [331], gastrointestinal [332], and urinary tracts [333], as well as the testis [334] and keratinocytes [335]. HBD-1 to -3 have activity against Gram-negative bacteria (e.g., *P. aeruginosa*, *E. coli*) and yeasts (e.g., *Candida albicans* and *Malassezia furfur*), with HBD-3 also having activity against Gram-positive bacteria (*S. pyogens*, *S. aureus* [including MDR *S. aureus*], and vancomycin-resistant *E. faecium*). Chimeric and synthetic HBD-4 are active against *E. coli*, *B. cepacia*, *P. aeruginosa*, *S. pneumoniae*, *S. aureus*, and *S. carnosus* [334]. θ-defensins are expressed in some Old World monkeys and orangutans, but not in New World primates or humans [336]. They are structurally dissimilar to α- and β-defensins and contain a macrocyclic backbone which is synthesized by the joining of two truncated α-defensins [337]. θ-defensins are active against *B. anthrax*, *S. aureus*, and *C. albicans* [338,339,340].

## 3. Structural and Physicochemical Properties of AMPs

Naturally produced AMPs are 10–100 amino acid residues long, with a majority less than 50 amino acids [341]. The shortest peptides in the Antimicrobial Peptide Database (APD), F3 and Gageotetrin A, consist of only two amino acids [342,343]. AMP length is cirtical for antimicrobial and membrane lytic activity because tendency to form secondary structures such as α-helices and β-sheets, which are essential for antimicrobial activity, reduces as the peptide length decreases [344,345,346].

Most AMPs are positively charged cationic peptides containing hydrophilic and hydrophobic residues at either end (i.e., amphipathic) [347]. Given that the surface of bacterial membranes are often negatively charged [348], positively charged AMPs initially bind to the membrane surface via electrostatic interactions. Once bound, the hydrophobic ends insert into the lipid bilayer causing membrane disorganisation by inducing toroidal pore (i.e., wormhole), barrel-stave, or the carpet model phenomenon (discussed further in Section 4.2), and, eventually, cell death [349,350,351,352]. The most positively charged peptides (net charge of +30) are Oncorhyncin II [353] and Oabac11 [354], with the most negatively charged (net charge −12) being cattle chrombacin [355]. The anionic AMPs likely form oligomers in the presence of Zn^2+^ and Ca^2+^ ions, enabling them to insert their lipid tails into the membrane [356,357,358].

AMPs can be classified according to their hydrophobicity, i.e., the proportion of hydrophobic amino acids [359]. Peptides lacking hydrophobic residues generally lack strong attachment to membranes, while those with high hydrophobicity such as gramicidin tend to reside longer in membranes [360]. While adsorption, membrane rupture, and antibacterial activity may occasionally be enhanced by increasing the positive charge on the AMP, under physiological conditions where ionic strength is high, highly charged and hydrophilic peptides lose much of their membrane lysis activity due to electrostatic screening [361]. Such inactivation of AMPs may be prevented by augmenting the hydrophobicity [362].

A majority of AMPs undergo PTMs, which significantly alters their structure. Such modifications enable binding to different targets including plasma membranes, nucleic acids, and proteins and/or allows the AMP to retain activity in a variety of environments [363]. A few AMPs undergo chemical modifications at more than one site. For example, styelin D, a 32-residue AMP from hemocytes of the subtidal ascidian tunicate *Styela clava*, undergoes extensive PTM. This AMP contains two unique amino acids (dihydroxyarginine and dihydroxylysine) and two unusual amino acids (6-bromotryptophan and 3,4-dihydroxyphenylalanine), and undergoes halogenation of tryptophan at position 2 and hydroxylation at various amino acids (Arg, Lys, Tyr) [364]. These modifications allow styelin D to retain activity at high salinity or low pH [364]. Artificially induced modifictaions may also alter key AMP properties. For example, cyclization of melittin resulted in a relatively small decrease in the membrane binding affinity of the cyclic analogue but increased antibacterial activity compared to the linear counterpart [365]. PTM may also be used to inactivate a peptide. For example, peptidyl arginine deiminase (PAD)-mediated citrullination of the human cathelicidin peptide LL-37 reduces its endotoxin neutralizing ability [366], while ADP-ribosylation (which considerably reduces the cationicity of LL-37) and carbamylation markedly reduce its biological activity [367,368].

### 3.1. Sequence Based Classification

The universal classification (UC) system categorizes AMPs into four classes based on covalent bonding patterns, namely UCLL, UCSS, UCSB, UCBB [369]. Developed at a time when the 3D structure of very few AMPs was known, this classification system does not take the 3D structure, source, or activity of AMPs into consideration [369].

#### 3.1.1. UCLL/Class L

**Figure 2:**
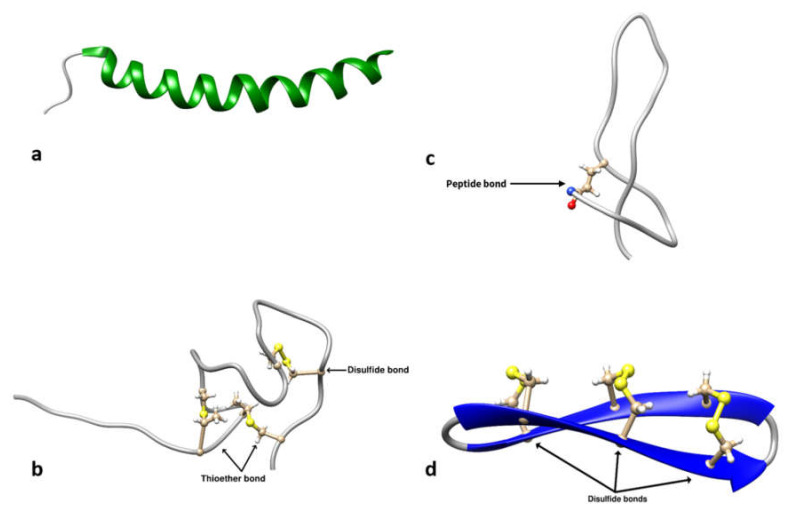
(**a**) UCLL (LL-37, amino acid sequence LLGDFFRKSKEKIGKEFKRIVQRIKDFLRNLVPRTES, PDB entry: 2K6O). **b**) UCSS (bovicin HJ50, amino acid sequence ADRGWIKTLTKDCPNVISSICAGTIITACKNCA, PDB entry: 2M8V). (**c**) UCSB (microcin J25, amino acid sequence GGAGHVPEYFVGIGTPISFYG, PDB entry: 1Q71). (**d**) UCBB (rhesus theta defensin-1, amino acid sequence GFCRCLCRRGVCRCICTR, PDB entry: 2LYF). Specifically, dilsufide and thioether bonds in (**b**,**d**) are represented by ball-and-stick model, with sulfur, carbon, and hydrogen atoms indicated in yellow, beige, and white, respectively; peptide bond in (**c**) is represented by ball-and-stick, with nitrogen, oxygen, carbon, and hydrogen atoms indicated in blue, red, beige, and white, respectively.

Class I (UCLL) contains linear AMPs such as LL-37 (Figure 2a) and magainins, which may be chemically modified (e.g., via amidation or glycosylation) at their side-chains or backbones [369]. Class I peptides are further categorized into two sub-classes based on the number of polypeptide chains, with linear single-chain AMPs additionally subcategorized into two families based on chemical modifications, namely UCLL1A unmodified peptide families that are amino acid-rich (e.g., Pro-Arg-rich PR-39), and non-amino acid-rich families (e.g., LL-37). The modified AMPs are further classified into two categories depending on the site of chemical modifications, namely UCLL1B the side chain (e.g., Piscidin 4, datucin, MccC7, heliocin) or UCLL1C the backbone (e.g., Aurein 1.2, bombinin H4, cypemycin (Linaridins), temporin A, and gramicidin).

#### 3.1.2. UCSS/Class S

Class II (UCSS) consists of AMPs that form chemical interactions between side chains [369]. These sidechain–sidechain interactions may occur within a single peptide chain or between two different peptide chains. Prominent members of this class include defensins (disulfide-bridged) and lantibiotics (thioether-bonded). Class II AMPs are further classified based on the number of interactions (chemical bonds) and polypeptide chains [369]. Those containing a single chain are the defensins, defensin-like AMPs, and lantibiotics, whereas those containing two chains include centrocin, lacticin-3147, distinctin, halocidin, and dipeptide lantibiotic Smb. Single-chain lantibiotics are further classified into several types based on the number of thioether bonds present. For example, there are two thioether bonds in bovicin HJ50 (Figure 2b), three in lacticin 481, four in cinnamycin, five in nisin and Subtilin, six in Paenicidin A, and seven in Geobacillin I [360,369].

#### 3.1.3. UCSB/Class P

Class III (UCSB) AMPs contain polypeptide chains with chemical interactions between the side chain of one amino acid and the backbone of another amino acid of the same chain [369]. Examples include lassos, which have a Glu8 or Asp9 residue covalently bonded to the amine terminus [370], and microcin J25, which contains a loop structure due to an interaction between the amine backbone of Gly1 and side chain of Glu8 [371]. Daptomycin also belongs to this class. Class III can be additionally divided based on the bond type formed [369]. For example, microcin J25 contains a CO-NH amide (Figure 2c), fusaricidin A contains a CO-O ester, and thuricidin CD a Cβ-S-Cα linkage.

#### 3.1.4. UCBB/Class O

Class IV (UCBB) contains circular AMPs that form a peptide bond between the amino and carboxyl termini of the polypeptide chain [369]. Additional modifications such as disulfide bonds may also be present. These AMPs have been isolated from bacteria (enterocin AS-48), plants (cyclotides), and primates (θ-defensins) (Figure 2d). Class IV AMPs are categorized based on additional linkages and the number of chemical bonds formed within the polypeptide chain [369]. For example, bacterial subtilosin A has a sidechain–backbone interaction (Cβ-S-Cα) while plant cyclotides and primate θ defensins have sidechain–sidechain interactions (Cβ-S-S-Cβ) [360,369].

### 3.2. Structure-Based Classification

Structure-based classification divides AMPs into four distinct groups based on the types of secondary structures present, namely (i) α-helical, (ii) β-sheets (at least two), (iii) αβ, and (iv) non-αβ [369].

#### 3.2.1. α-helix AMPs

**Figure 3:**
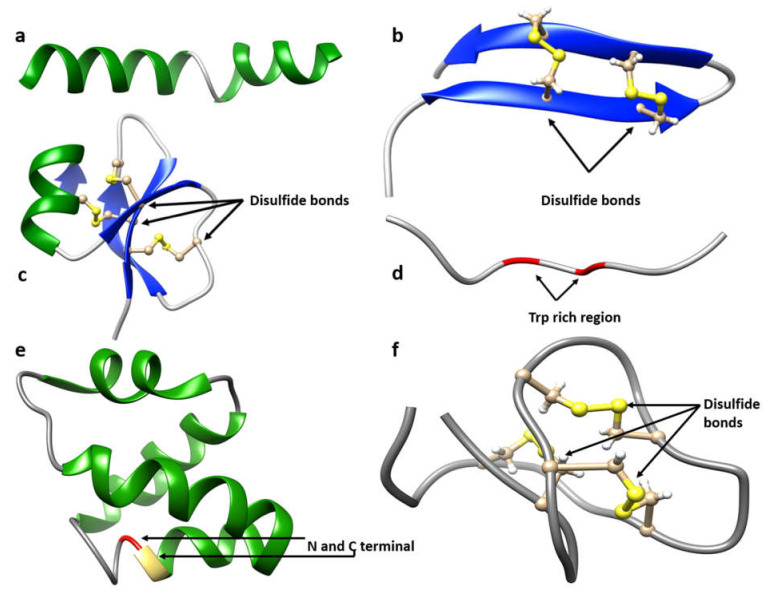
Representative AMPs from five structure-based classes. (**a**) α-helix AMPs (melittin, amino acid sequence GIGAVLKVLTTGLPALISWIKRKRQQ, PDB entry: 2MLT). (**b**) β-sheet AMPs (protegrin-1, amino acid sequence RGGRLCYCRRRFCVCVGR, PDB entry: 1PG1). (**c**) αβ-AMPs (hBD2, amino acid sequence GIGDPVTCLKSGAICHPVFCPRRYKQIGTCGLPGTKCCKKP, PDB entry: 1FD4. (**d**) non-αβ AMPs (indolicidin, amio acid sequence ILPWKWPWWPWRR, PDB entry: 1G89); Trp-rich regions are indicated in red. (**e**) Cyclic AMPs with no thioether nor disulfide bonds (carnocyclin A, amino acid sequence LVAYGIAQGTAEKVVSLINAGLTVGSIISILGGVTVGLSGVFTAVKAAIAKQGIKKAIQL, PDB entry: 2KJF). The *N*- and *C*-terminal linkage is represented by red and beige, respectively. (**f**) cyclic AMPs with thioether or disulfide bonds (kalata B1, amino acid sequence NGLPVCGETCVGGTCNTPGCTCSWPVCTR, PDB entry: 1K48). Disulfide bonds are represented by ball-and-stick model with sulfur, carbon, and hydrogen atoms indicated in yellow, beige, and white, respectively.

These are the most studied structures [372], with cecropin, pleurocidin, melittin (Figure 3a), magainin, and moricin being the best described [373]. The α-helical AMPs are the most abundant in nature and have been isolated from numerous species including plants, insects, amphibians, fishes, and mammals. Several studies have revealed that the α-helical structure of these AMPs is highly reliant on the interaction with the targeted membranes [372,374,375]. This conformational change upon interaction segregates the hydrophilic residues from the hydrophobic residues, with the peptide assuming an amphipathic structure essential for membrane-targeting activity [376]. The structure and activity of amphipathic α-helices is well characterized by a typical barrel-stave model that forms a transmembrane pore. The α-helices form bundles in the membrane where the hydrophobic region interacts with the membrane lipid core and the hydrophilic region points inward, resulting in a pore [377]. The α-helices are often rich in Leu, Ala, Gly, and Lys.

#### 3.2.2. β-sheet AMPs

β-sheet AMPs consist of at least two β-strands with many linear structures adopting a β-hairpin-like conformation [168]. Most members of this family contain conserved cysteine residues that form disulfide bridges critical to their conformation and functions [378]. For example, the disulfide bonds in defensins provide structural stability and reduce protease-mediated degradation [379]. Antimicrobial activity is usually attributed to the cationic residues and hydrophobic side chains exposed on the antiparallel β-sheets. This class of AMPs include protegrin-1 (PG-1) (Figure 3b) [380], thanatin [381], tachyplesin [382], polyphemusin I [383], and gomesin [384]. Defensins constitute the major group of β-sheet AMPs and can be further classified into subfamilies based on the location of disulfide bonds. This group of peptides are often rich in Leu, Ala, Gly, and Lys.

#### 3.2.3. αβ AMPs

This class of AMPs contains both α-helices and β-sheets, and strongly target membranes [369]. The most prominent members are the plant and insect defensins that have antifungal activity due to interactions with fungal membrane sphingolipids or microsomal membranes [385,386]. The antifungal plant-derived peptide pisum sativum defensin 1 (Psd1) contains a βαββ fold that interferes with cyclin F in *Neurospora crassa*, thereby affecting the cell cycle [387,388]. RsAFP2, a defensin from *Raphanus sativus* that interacts with glucosylceramides of yeast and fungi, activates a signaling pathway in *C. albicans* involved in reactive oxygen species, resulting in cell death [389]. Other class members have alternative targets. Plant defensin Nad1 binds to membrane phosphatidylinositol 4,5-bisphophate before interacting with intracellular targets, resulting in the accumulation of reactive oxygen species [390,391]. Examples of αβ-AMPs in humans are the beta-defensins hBD1, hBD2 (Figure 3c), and hBD3, which contain an αβββ fold [379].

#### 3.2.4. Non-αβ AMPs

Non-αβ AMPs, also called extended or loop peptides, lack both α-helix and β-sheet structures and are classified as tryptophan-rich, proline-rich, and glycine-rich peptides [360].

Many tryptophan-rich peptides have an amphipathic conformation. Indolicidin (Figure 3d) has an amphipathic structure that consists of a central tryptophan (Trp)-rich region essential for peptide anchorage. Indolicidin bound to dodecylphosphocholine (DPC) micelles showed an interaction between the Trp6 and Trp9 aromatic rings packed against Pro7 and Pro10, respectively [392,393]. Similarly, tritrpticin in sodium dodecyl sulfate (SDS) micelles contain an amphipathic turn that is clustered with three tryptophan residues [394]. In lactoferrin B_2_, the Trp-rich regions form a non-αβ conformation with a deformed backbone upon interacting with SDS micelles [395].

The proline-rich peptides are 15–39 residues long [396] and act on intracellular targets [397,398]. Recent studies have shown that these peptides adopt a non-αβ structure that blocks the ribosomal tunnel, thereby preventing aminoacyl tRNA entry into the A-site [399]. Glycine-rich peptides are found in a variety of insect species and typically have a molecular weight ranging from 8 (holotricin) to 30 (sarcotoxin II) kDa [373]. KAMP-19, a glycine-rich peptide from the human eye, possesses a non-αβ structure that deforms bacterial cell envelopes and induces pore formation [400].

#### 3.2.5. Cyclic and Unusual or Complex AMPs

Based on the special structural features discussed in Section 3.1 (UCBB/class O) [369], these peptides can be grouped as a fifth class of AMPs [401]. Furthermore, this group can be subclassified based on cyclic topology (either head-to-tail or head-to-side-chain) and crosslinks (e.g., thioether or disulfide bonds) [401]. Cyclic bacteriocins (MW ~6 kDa) are a group of ribosomally synthesized peptides that are characterized by their *N*- to *C*-terminal covalent linkage and lack of additional linkages [402]. For example, carnocyclin A isolated from *Carnobacterium maltaromaticum* UAL307 and enterocin NKR-5-3B isolated from *Enterococcus faecium* NKR-5-3 consist of four α-helices and have their *N*-terminal linked to the *C*-terminal. In carnocyclin A, the *N*-terminal Leu at postion 1 is linked to *C*-terminal Leu at position 60 (Figure 3e), whereas, in enterocin NKR-5-3B, the *N*-terminal Leu at position 1 is linked to the *C*-terminal Trp at position 64. [403,404]. Other backbone-cyclized peptides consist of additional intramolecular thioether and disulfide bonds to stabilize structures. For example, mammalian θ-defensin RTD-1 (Figure 2d) and plant cyclotide Kalata B1 (Figure 3f) are cyclic AMPs containing three disulfide linkages that form a cysteine-knotted framework responsible for conferring significant structural stability to the peptides as compared to linear peptides [401,405,406]. The anti-HIV activity of Kalata B1 is due to an intact cyclic backbone [407]. The cysteine knot is a structural motif forming an embedded ring by three disulfide bonds in which the connecting backbone segments of two disulfide bonds are threaded by a third disulfide bond [408]. This cysteine knot framework can tolerate a wide range of amino acid substitutions and has shown great promise as a scaffold in drug design and protein engineering [405,408]. For example, circulin A and B are macrocyclic cylotides belonging to the bracelet sub-family. The disulfide bond order for circulin A and B is Cys1-Cys17, Cys5-Cys19, and Cys10-Cys24; this arrangement forms a compact structure and fold that is stabilized by an extensive network of hydrogen bonds [409,410]. They exhibit anti-viral activity [411] and can be considered as potential anti-HIV drugs [115]. Tachystatin B is an antimicrobial peptide with three disulfide bonds between Cys4-Cys20, Cys11-Cys25, and Cys19-Cys37. The Cys19-Cys37 disulfide bond traverses through the closed ring formed by the two other disulfide bonds and two segments of the backbone (Cys4-Cys11 and Cys20-Cys25), forming an inhibitory cysteine-knot motif that is considered essential for antimicrobial activity [412]. Subtilosin A is a prominent example of unusual cyclic AMP as it has an amide bond between the *N*- and *C*-termini and three cross-links between the sulfurs of Cys13, Cys7, and Cys4 and the α-positions of Phe22, Thr28, and Phe31, respectively [413]. A detailed review on the knot motif in cyclic AMPs can be found elsewhere [408].

## 4. Diverse Activities and Modes of Action

AMPs are characterized based on their target organisms and mechanisms of action.

### 4.1. Antiviral AMPs

Antiviral AMPs possess diverse mechanisms of action against both RNA and DNA viruses and can be categorized into the following types based on their mode of action: (i) viral membrane targeting AMPs (e.g., indolicidin, human α-defensin 1) that eliminate viruses by incorporating themselves into the viral envelope, thereby creating membrane instability and rendering the virus incapable of infecting the host cell [414,415]; (ii) viral adsorption-targeting antiviral AMPs that act by binding to specific viral receptors on target cells, thereby inhibiting viral binding and subsequent entry [416]. For example, defensins interact with herpes simplex virus (HSV) glycoproteins to prevent viral attachment to host receptors [417]. Heparan sulfate is a well-studied example of a negatively charged host cell surface receptor required for HSV entry. Lactoferricin, derived from the cleavage of lactoferrin, inhibits herpes infection by occupying heparan sulfate receptors as well as viral particle receptors, thereby blocking virus adsorption and entry [418,419,420]. (iii) AMPs targeting intracellular components; for example, NP-1, an alpha-defensin from rabbit neutrophils, inhibits Herpes simplex virus type 2 (HSV-2) by blocking viron protein VP16, which is essential for viral translocation into the nucleus [421,422]. Melittin and Cercopin A are insect-derived AMPs that exhibit anti-HIV1 (type 1 human immunodeficiency virus) activity by interfering with viral transcription [423]. Cecropin A can also inhibit the Junin virus (JUNV) by targeting viral nucleocapsid *N*-protein biosynthesis [424]. Red fluorescent proteins (RFP) from *Bombyx mori* (Silkworm) can disrupt viral nucleocapsids, thereby inhibiting replication [425,426,427]. L4-1, a peptide isolated from silkworm faeces, produces reactive oxygen species in visible light that damage viral proteins, conferring marked antiviral activity against enveloped viruses (e.g., HJV [Sendai virus], HSV1, and HIV1 [human immunodeficiency virus type 1]) but not non-enveloped viruses [428]. Indolicidin, in addition to the mechanism of action mentioned above (Section 2.5.4), inhibits arenavirus replication by interfering with late-phase events such as viral morphogenesis and the inhibition of viral release from the host cell [424].

### 4.2. Antibacterial AMPs

Antibacterial AMPs are the most thoroughly investigated AMP class, with a majority being cationic and amphipathic. They interact with anionic bacterial membranes, causing disruption to the lipid bilayer [429,430]. Certain anionic peptides also have antibacterial activity that includes surfactant-associated anionic peptides (SAAP), frog maximin-H5, and human dermcidin [431,432].

Certain lipopetides (e.g., polymyxins B and E, daptomycin) and glycopeptides (e.g., vancomycin, teicoplanin, telavancin, dalbavancin, and oritavancin) are currently available in the clinic. Both polymyxins have similar in vitro potencies and a spectrum of activity against primarily Gram-negative pathogens, including many responsible for MDR nosocomial infections [433]. Daptomycin is a 13-residue cyclic lipopeptide with a hydrophilic core. It was isolated from *Streptomyces roseosporus* and is used for the treatment of recalcitrant Gram-positive infections [434]. The glycopeptides have a broad antibacterial spectrum against Gram-positive bacteria [435]. Bacterial killing by the glycopeptides is due to their binding to cell wall precursors rather than acting directly on an enzyme active site. For example, the cyclic heptapeptide core of vancomycin forms a unique binding pocket for the D-alanine (D-Ala) dipeptide D-Ala-D-Ala located at the *C*-terminus of the pentapeptide precursor, inhibiting peptidoglycan chain formation and cross-linking [436,437].

Antibacterial AMPs are subcategorized into two types based on their mechanisms of action: (i) membrane disrupting and (ii) non-membrane targeting peptides [438]. However, some AMPs may act via both mechanisms.

#### 4.2.1. Membrane Targeting AMPs

**Figure 4:**
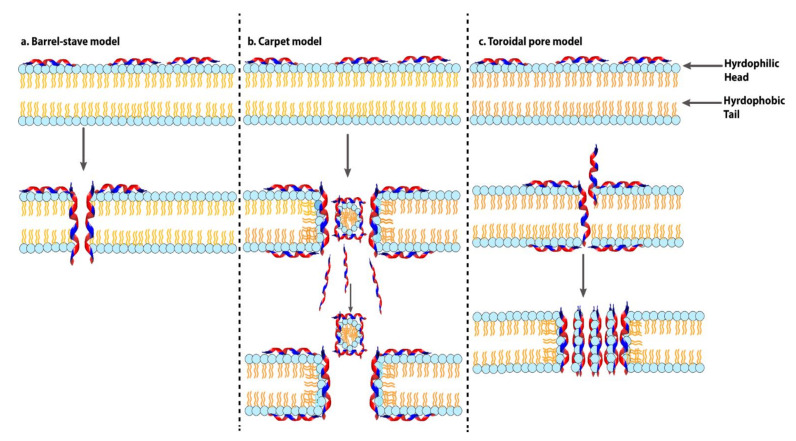
(**a**) Proposed barrel-stave model [443], (**b**) carpet model [444], and (**c**) toroidal pore model [445]. The hydrophilic and hydrophobic regions of the AMPs are represented in blue and red, respectively. The hydrophilic head and hydrophobic tail are represented in cyan and orange, respectively.

Many antibacterial AMPs target bacterial cell membranes via initial electrostatic interactions between positively charged peptide molecules and the negatively charged cell surface, followed by hydrophobic interactions between the peptide amphipathic domain and the membrane phospholipids [375]. The modes of action proposed for subsequent pore formation are the barrel-stave [439], carpet-like [440], toroidal pore (Figure 4) [441], aggregated channel [393], and clustering of anionic lipids models [442]. Some AMPs act via more than one mechanism.

The barrel-stave mechanism is characterized by the vertical aggregation of helices into the lipid bilayer. The transmembrane peptides bundle similarly to the staves of a barrel, with their hydrophobic face aligned with the central lipid region of the lipid bilayer while the hydrophilic peptide constituents form the inner water-filled pore region [446]. The stable channels (barrel-like pores) formed in the cell membrane result in cytoplasmic outflow and, in severe cases, membrane collapse and ultimately cell death [447]. This mechanism is exhibited by pardaxin [448], alamethicin [449], ceratotoxin [450], δ-endotoxin [451], peptaibols such as antiamoebin I (AamI) [452] and Class II bacteriocins [453], some magainins, PGLa [454], and MSI78 [455].

In the toroidal pore model, the peptides inserted into the membrane cause a continuous bending of the lipid monolayer from top to bottom [449]. The central water core is lined with the embedded peptides and lipid head groups. During the formation of a toroidal pore, the polar regions of the peptides align with the lipid polar head groups. The toroidal pore mechanism is similar to the barrel-stave model but differs in the fact that peptides are aligned with the lipid head groups even when they are inserted perpendicularly into the lipid bilayer [446], the pores formed are transient, and the structures formed are less stable than barrel-stave formations [456]. Examples of peptides that act via this mechanism include arenicin, magainin 2, and lacticin Q [457,458,459]. Melittin commonly acts via toroidal pore formation, although barrel-stave or detergent mechanisms have also been suggested [449,460,461]. AMPs maculatin1.1, protegrin-1, tritrpticin, and pleurocidin act by forming ion channels via the toroidal pore model whereby AMPs bind to cell membrane surface phospholipids, form peptide-lipid polymers, and eventually enter the cell [462,463,464].

The carpet or detergent-like model was first described for dermaseptin S [351]. It hypothesizes that the peptides initially aggregate on the membrane in monomeric or oligomeric form (covering the membrane like a carpet), with the hydrophobic regions subsequently interacting with the cell membrane and the hydrophilic ends facing the aqueous solution. When a concentration threshold is reached, aggregation of the peptides induces membrane permeation with subsequent membrane disruption [430]. Other examples of peptides that likely act via the carpet-like model are cecropins [465], indolicidin [393,466,467], aurein 1.2 [468], caerin 1.1 [469], and trichogin GA IV [470].

Anionic lipid clustering activity involves the preferential interaction of cationic AMPs with anionic charged lipids, causing lateral segregation of these lipids from zwitterionic ones, resulting in the formation of phase boundary defects between lipid domains [442]. Such activity is exhibited by MSI-103, PGLa, Magainin, KIGAKI, MAP, and penetratin [442].

#### 4.2.2. Non-Membrane Targeting/Intracellular AMPs

Some AMPs can kill bacteria without affecting membrane stability. These AMPs directly penetrate bacterial cells and interfere with essential cellular activities including DNA replication, transcription, translation, protein folding, and cell division [471,472]. Nucleic acid-targeting AMPs include the buforins I and II [473,474], which have been shown to penetrate the cell membrane of *E. coli* without permeabilization [475], subsequently binding to DNA and RNA [473,474,475]. Parasin I and hipposin from catfish skin mucus exert their antimicrobial activity via a similar mechanism to buforins [476,477]. At high concentrations, indolicidin, a Trp/Pro-rich AMP of 13 residues, induces membrane permeabilization, which allows peptides to continuously enter the cytoplasm where they interfere with DNA synthesis [478]. Specifically, indolicidin targets the abasic site of DNA causing crosslinks with single or double-stranded DNA, as well as inhibiting DNA topoisomerase I [467]. Other DNA-targeting AMPs include ostricacin I (OSP1) and ostracacin 2 (OSP2), oabac 5mini, and microcin B17 (the latter blocking DNA gyrase) [479,480]. PR-39 acts by secondary DNA synthesis inhibition by targeting DNA replication-associated proteins [481]. Cell division-blocking AMPs act via inhibiting DNA replication and DNA damage responses (e.g., the SOS response), thereby blocking the cell cycle or inducing failure of chromosome separation [482]. Microcin J25 arrests cell division in *E. coli* by targeting RNA polymerase (RNAP) [483]. Human α-defensin 5 interferes with cell division in Gram-negative bacteria by bleb formation, cellular elongation, and clumping [484].

Protein synthesis-targeting AMPs exert their antibacterial effect by blocking protein biosynthesis either through effects on transcription, translation, or protein assembly [485]. For example, Bac7 (1–35) inhibits translation by interfering with ribosomes (Figure 3) [398]. Pleurocidin, in addition to ion channels formation via the toroidal pore model, likely inhibits protein biosynthesis in *E. coli* [486]. The hybrid peptide DM3 exhibited broad spectrum, rapid antibacterial killing via the disruption of DNA replication, transcription, ribosome assembly, and amino acid biosynthesis [487]. Apidaecin has recently been shown to competitively bind with release factors on the A-site of ribosomes, inhibiting the termination step of translation [488]. Human neutrophil peptide defensin (HNP)-1 sequentially induces membrane permeabilization of the outer and inner membranes in *E. coli* as well as inhibition of DNA replication, transcription, and protein synthesis [324]. Lactoferrin B, PR39, P-Der, and Bac7, when incubated with *E. coli*, demonstrated inhibitory activity on arginine decarboxylase and various other proteins [489]. Defensins and dermaseptins also arrest protein synthesis [490].

Protein folding inhibition occurs with some insect-derived, proline-rich peptides known to inhibit bacterial DNA replication by interfering with protein folding. Pyrrhocoricin can block the molecular chaperones DnaK and GroEL, and also reduce DnaK ATPase activity via competitive inhibition [491,492]; drosocin (DnaK and GroEF) [491] and apidaecin (DnaK) [493] exert similar inhibition. Similarly, Bac7 (1–35) potentially binds DnaK, blocking the protein folding of molecular chaperones (DnaK-DnaJ, GrpE-ATP) in a dose-dependent manner [494]. Oncocin, another proline-rich peptide of 19 residues, exerts activity against Gram-negative pathogens such as *E. coli*, *P. aeruginosa*, and *A. baumannii* by freely penetrating (without lysing) the cell membrane and subsequently binding with DnaK, thereby interfering with protein folding [495,496].

Other AMPs act via inhibition of protease activity, thereby interfering with critical cellular metabolism. For example, eNAP-2 from equine leukocytes exerts antibacterial activity against *E. coli*, *P. aeruginosa*, *S. zooepidermicus*, and *K. pneumoniae* by preferentially binding bacterial serine protease, proteinase K, or subtilisin A to form non-covalent complexes [497]. Ixodidin is a cysteine-rich, 65-residue AMP from tick hematocytes that inhibits the cellular metabolism by blocking elastase and chymotrypsin [498]. Histatin 5, from the salivary secretions of human submandibular and parotid glands, is a histidine-rich, cationic AMP of the histatin family [499]. Against *S. mutans*, a major cause of dental caries [500], histatin 5 blocks host as well as bacterial proteases, preferentially binding to trypsin-like proteases [501].

Cell wall-inhibiting AMPs target lipid II, an essential constituent of peptidoglycan. Nisin, a lantibiotic and the best-characterized AMP of this class, possesses lipid II sequestering activity and inhibits the transglycosylation step in cell wall biogenesis [69]. Mersacidin, a globular lantibiotic possessing four thioether bridges, binds to lipid II and inhibits transglycosylation in a similar manner to nisin (Figure 3) [502]. Similar activity has been observed for lacticin 481 and cinnamycin (both lantibiotics) [503], HBD3 and HNP1 [504,505], and the novel fungal AMP copsin [147].

### 4.3. Antifungal AMPs

Antifungal peptides have been isolated from a number of species of archaea, bacteria, plants, and animals [506]. The fungal cell wall is mainly composed of chitin [507]. Antifungal AMPs possess similar mechanisms of action to antibacterial AMPs, namely the (i) barrel-stave (e.g., observed with Amphotericin B which binds to membrane ergosterol) [508,509], (ii) carpet-like (e.g., Dermaseptin, disrupting microbial cell membranes) [510,511], and (iii) toroidal pore (e.g., LL37 interacts with the cell wall carbohydrates of *candida* and protegrin-1) [512] models. Other mechanisms of action include (iv) inhibition of 1,3-β-glucan biosynthesis (e.g., echinocandins, pneumocandins, aculeacins), (v) inhibition of chitin biosynthesis (e.g., aureobasidins), and (vi) interference with other critical intracellular targets such as DNA-targeting actinomycins that intercalates DNA and buforins that target the DNA. Examples of this latter group include the tridecapeptide indolicidin, which interferes with DNA processing enzymes and repair mechanisms, and VL-2397 (from *Acremonium persicinum*), which acts as an iron-chelating siderophore that causes hyphal elongation arrest [242,513]. The structures of most antifungal AMPs are currently not well determined. However, upon interacting with membranes, some naturally occurring AFPs assume α-helical, β-sheet or hairpin (with two cysteine residues) structures, or mixed α-helix/β-sheet conformations. For further information on antifungal AMPs, we refer interested readers to the reviews by Fernández de Ullivarri et al. and De Cesare et al. [506,513].

## 5. AMP Databases

**Figure 5:**
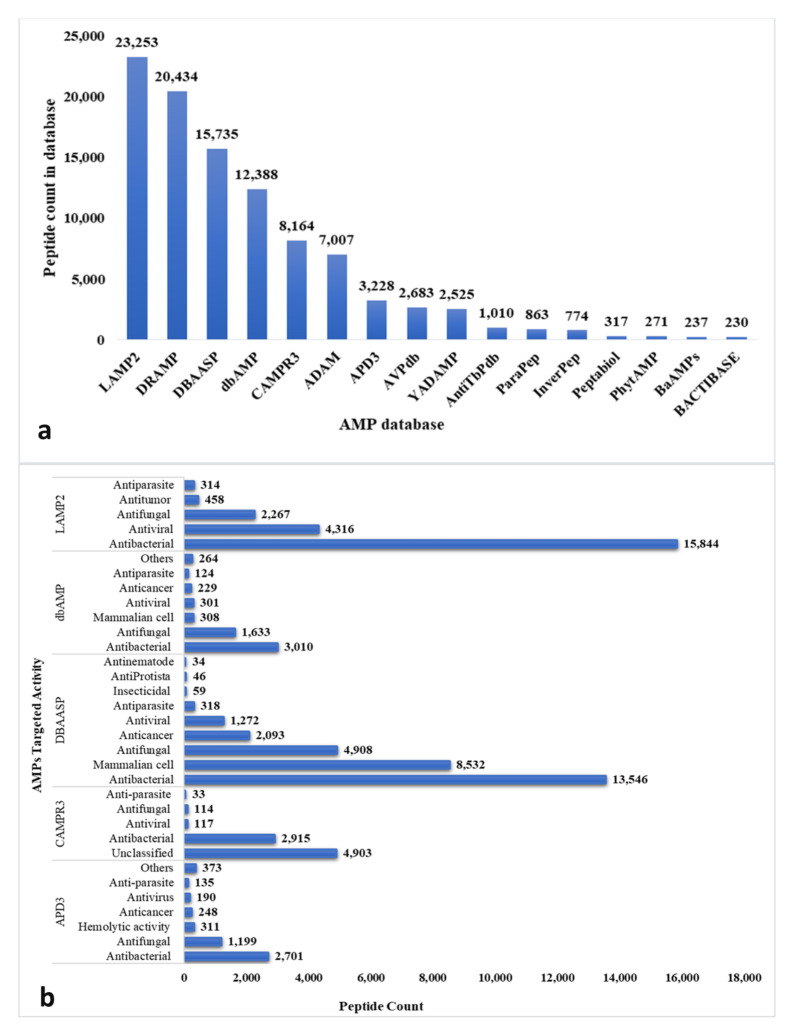
(**a**) The total number of AMPs contained in a variety of databases and (**b**) breakdown of AMPs by targeted activity in five representative databases.

Leveraged by the progress of systems pharmacology, chemical biology, and computational biology, the number of naturally produced and chemically synthesized AMPs in databases has rapidly increased in recent years. Of the AMPs catalogued, antibacterial peptides represent the largest group (Figure 5). Here, we summarize the major regularly curated databases and their associated unique computational tools for AMP discovery and engineering. Some important prediction functionality tools found in many of these databases are briefly described separately in Section 6.

### 5.1. APD3

The Antimicrobial Peptide Database (APD3; available at https://aps.unmc.edu/) is one of the largest databases [234] (last accessed on 2 October 2020). It has catalogued 3250 AMPs, including 2409 from animals, 360 from plants, and 365 from bacteria.

**Figure 6:**
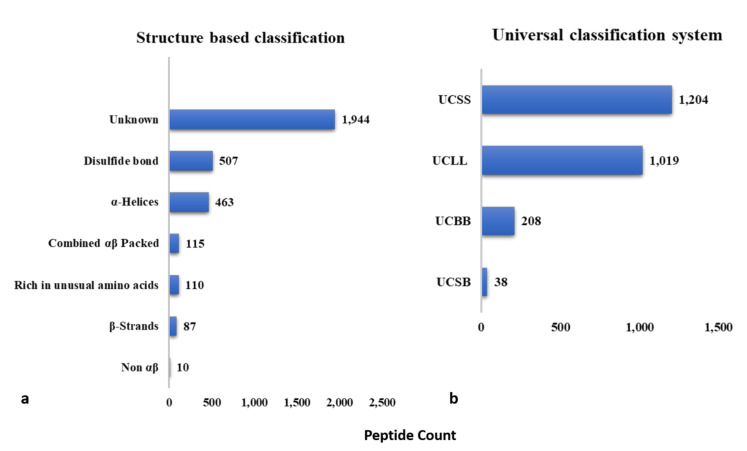
(**a**) Number of AMP 3D structures and (**b**) the AMP universal classification system. The x-axis represents the peptide count and, the y-aixs, the strucutre and class of AMPs.

The APD3 provides searchable annotations including source organism, peptide sequence, and PTM (24 chemical modifications are included) (Wang, 2015). Amidation was the most common PTM, followed by Rana Box (via a single S–S bond) and backbone cyclization. The AMP binding targets interface allows searching for the determined mode of action of an AMP, with 10 modes of actions incorporated. Peptide binding to membrane targets is the dominant mode followed by LPS and sugar/carbohydrates targeting. APD3 provides extensive structural classification of AMPs (Figure 6), with the 3D annotated structures deposited in the Protein Data Bank database (PDB) [514]. The structure-determining methods include nuclear magnetic resonance (NMR, 385 structures), circular dichroism (CD, 248 structures), and X-ray crystallography (58 structures). Users can search for AMPs according to the 3D or covalently bonded structures.

The APD3 database also provides 21 useful tools to predict, modify, and carry out extensive analyses of peptides. Tools include a prediction interface to analyse the potential of a given amino acid sequence to form an AMP. The submitted query additionally returns information such as the amino acid percentage and composition, hydrophobicity content, total net charge, molecular weight, chemical formula, grand average of hydropathicity index (GRAVY, representing the hydrophobicity of a peptide), and Boman index (BI, estimating the protein-binding potential), along with structural information based on the amino acid composition. The peptide improvement tool can be used to increase the potency of peptides. The APD3 is updated frequently, with the latest news and facts provided in the What’s New interface.

### 5.2. CAMP_R3_

The Collection of Anti-Microbial Peptides database (CAMP_R3_; available at http://www.camp3.bicnirrh.res.in (accessed on 2 October 2020)) was developed to promote AMP family-based studies. The database is divided into four main sections, namely (i) sequences (currently 8164 AMP sequences), (ii) structures (757 structures), (iii) patents (2083 patents), and (iv) signatures (36 patterns and 78 Hidden Markov Models (HMMs)). The CAMP_R3_ database has classified the collected AMPs into 45 different families based on signatures acquired from HMMs and patterns. Nine different tools are incorporated into the database, most notably (i) AMP Prediction tools for predicting AMPs from amino acid sequences, detecting antimicrobial regions in peptides, and rational design/improvement of AMPs; (ii) CAMPSign feature searches for peptide patterns related to 45 families present in the database; (iii) Vector Alignment Search Tool (VAST) to identify distant homologs based on 3D geometrical criteria; (iv) PRATT for identifying conserved patterns in sets of protein sequences; (v) ScanProsite to search input sequences against Prositemotif; (vi) Pattern Hit Initiated (PHI) BLAST for pattern searches in protein sequences; (vii) JackHmmer for distant homology detection.

### 5.3. dbAMP

The dbAMP (available at http://csb.cse.yzu.edu.tw/dbAMP/ (accessed on 2 October 2020)) is an integrated database that collects AMPs from public databases and the literature [515]. Currently, it consists of 12,389 AMPs of which 4270 have been validated and 8118 predicted. Specific AMPs can be retrieved by AMP ID or specific amino acid sequences. Functional type icons are provided to narrow down the search options. The dbAMP provides a unique tool to mine AMP cryptic regions from transcriptomics or proteomics data. Specifically, next generation sequencing (NGS) detection enables the user to detect critical regions of antimicrobial potency from the metatranscriptomics analysis of transcriptomics or proteomics data.

### 5.4. DBAASP

The Database of Antimicrobial Activity and Structure of Peptides (DBAASP; available at https://dbaasp.org (accessed on 2 October 2020)) is a manually curated database that currently contains 17,532 entries [516]. A unique feature of the database is the molecular dynamics (MD) simulation models with trajectory files and self-consistency data for a large number of peptides. Currently, there are 5618 MD models in the database. These data can be used to better understand structure–activity relationships for rational peptide design. The property calculator tool enables evaluation of an AMPs physicochemical properties and provides six hydrophobicity scales based on the literature [359,517,518,519,520,521].

### 5.5. LAMP2

The Linking Anti-Microbial Peptides database (LAMP; available at http://biotechlab.fudan.edu.cn/database/lamp/index.php (accessed on 2 October 2020)) is an online repository for the discovery and design of AMPs [522]. The database contains 23,253 unique AMP sequences including 7824 natural and 15,429 synthetic AMPs of length shorter than 100 residues.

## 6. Prediction Functionality in AMP Databases

The post-genomic era has boosted AMP discovery and design. Sequence alignment and pattern matching have been intensively employed for the identification of unannotated AMPs. Recently, the explosive growth of sequencing data has stimulated the application of powerful machine learning algorithms in biomedical areas, including genomic mining and the design of AMPs [523]. Many AMP prediction tools have been developed and widely used, including FASTA [524], BLAST [525], HMM [526], REGEX [527], molecular dynamics simulations, and machine learning algorithms. Here, we summarize several major approaches for AMP prediction, namely sequence alignment, pattern-matching, molecular dynamics simulations, and machine learning algorithms (examples of the latter include support vector machine, artificial neural networks, and random forest).

### 6.1. Sequence Alignment

Sequence alignment is a method widely employed to determine homology in DNA and protein sequences [528]. The most representative tools for homology detection are BLAST (for pairwise alignment) [529] and CLUSTAL (for multiple sequence alignment) [530]. Wang et al. used BLASTP (searches protein databases against a query protein), the nearest neighbor algorithm, and a feature selection approach involving amino acid and pseudo-amino acid composition including codon diversity, electrostatic charge, polarity, molecular volume, and the secondary structure to create an AMP prediction tool [531].

### 6.2. Pattern-Matching

Pattern matching strategies are more efficient than the sequence alignment method, reducing computational time while simultaneously detecting more remote protein homologs. The Profile Hidden Markov Model (profile-HMM) is the most widely used pattern matching strategy [526].

### 6.3. Profile-HMM

The profile-HMM is a probabilistic, sensitive approach for detecting distant homology from multiple sequence alignments [526]. This approach generates a profile from a multiple sequence alignment and then subjects it to HMMER (a profile-HMM tool widely used in protein family databases such as Interpro and Pfam [532,533]) to determine the evolutionary events that took place in a set of related sequences.

A drawback of the profile-HMM approach is peptide promiscuity, namely the ability of an AMP to perform different functions when exposed to different environmental conditions [534]. As a conserved peptide detected by local alignment or pattern detection may not have antimicrobial activity [535], effective computational prediction tools for activity are needed. To this end, machine learning approaches have been applied in detecting potential AMPs.

### 6.4. Machine Learning and Deep Learning

Machine learning involves the construction of computer systems that employ artificial intelligence (AI) algorithms which automatically learn from data and improve through experience [536]. It can be classified into (i) supervised learning in which input data are labelled prior by the user to train the system [537], and (ii) unsupervised learning where no pre-existing labels are provided [538]. Supervised learning methods utilized in AMP prediction include artificial neural networks (ANN), support vector machines (SVM), quantitative matrices, random forests (RF), k-nearest neighbors (k-NN), and self-organized maps (SOM) [539]. Deep learning is a subfield of machine learning that structures algorithms in layers, creating an “artificial neural network”. It was recently used to accurately predict protein structures even in the absence of known similar structures [540].

Support vector machine (SVM) is a supervised learning method operating on a set of well-characterized vectors that learns classifiers used to classify data [541]. In this regard, a combined sequence alignment method utilizing Lempel–Ziv (LZ) complexity and SVM-pairwise algorithm which enables rapid and effective peptide prediction was proposed by Ng. et al. [542]. Random forest algorithm is based on the combination of decision trees from the feature vectors. The inference is made on votes cast by the trees [543], an example being the identification and prediction of antitubercular peptides using a combined RF and SVM algorithm [544]. For the prediction of lantibiotics, a combination of four different ML algorithms (SVM, Sequential minimal optimization (SMO), naïve bayes (NB), and RF) was implemented [545]. Deep learning algorithms have also been implemented to predict and identify AMPs. A multi-scale convolutional network model (deep neural network) outperformed existing state-of-the-art models when used for AMP discovery [546], and a long short-term memory (LSTM) generative model and bidirectional LSTM classification model were effective at generating novel antibacterial AMPs that could be utilized as new antibiotic leads [547]. SVM combined with deep learning-based features identified 436 possible antimicrobial proteins in the genome of *Helobdella robusta* [548]. Discriminant analysis (DA), which is a multivariate approach [549], quadratic discriminate analysis [550], and conditional random fields [551] may also be used for AMP prediction.

### 6.5. Molecular Dynamics (MD) Simulations

As the number of peptides discovered increases exponentially, studying their antimicrobial activity and mode of action is challenging. Use of MD simulations is a comparatively convenient method to investigate the activity and mode of action of AMPs [552]. Advancements in MD simulations have been driven by improvements in hardware, mathematical modeling, and algorithms, as well as the development of new force fields that better parameterize the chemical interactions. MD simulations at atomic resolution have been successful in determining peptide binding and folding, partitioning into lipid bilayers, and how the peptide channels that conduct ionic and other material across membranes form [553]. A few examples of recent successes using MD simulations include the rational peptide design approach to describe, at the microseconds scale, the interaction of indolicidin with membranes and design indolicidin analogues with enhanced antimicrobial activity and low hemolysis [554], and the determination of the structural stability and compactness of 37 lantibiotics using dynamics simulations [555]. The latter study revealed a lack of correlation between the structural and sequence diversity of lantibiotics, a property that could be explored to design novel, higher efficacy lantipeptides. The use of MD simulations in combination with prediction algorithms has also been used to improve the activity of peptides, resulting in the discovery of potent antibacterial AMPs [556]. Talandashti et al. used all-atom and coarse-grained MD simulations to gain molecular-level insights into pleuricidin pore-formation and its associated antimicrobial activity [557]. A similar process was used by Catte et al. to determine the mechanism of membrane interaction of chrysophsin-3 (chrys-3), a highly cationic peptide of 20 amino acids from the gills of red sea bream [558]. MD simulation studies have also been used to examine the energetics of melittin and its insertion mechanism in a mimic of a bacterial membrane (DOPC/DOPG mixed bilayer). The energy barrier results from MD were found to be consistent with the free energy estimation of melittin molecules [559]. MD simulations in combination with machine learning have been used in AMP prediction based on 3D descriptors as compared to the traditional 2D descriptors [560]. MD simulations have also played a role in demonstrating the conformation of single and multiple monomers of bombinin H2, the latter forming self-aggregated structures [561]. Similarly, the carpet-like mechanism of megin peptide was determined by MD in combination with spectroscopy and zeta potential [562]. The mechanism of protegrin-1 (PG-1) pore formation was also determined by multistep MD studies that revealed the insertion, translocation, and induction of the pore [563]. The differential interactions of LL37 with a mimick of bacterial (POPG) and mammalian (POPC) membranes were determined using MD studies [564]. Fengycin, a cyclic lipopeptide with antifungal activity, was shown to form stable oligomers in model fungal membranes using MD [565]. The implementation of a combined deep learning and MD simulation approach also resulted in the rapid discovery of AMPs with high potency against diverse Gram-positive and -negative pathogens (including MDR *Klebsiella pneumoniae*) [566]. While many other MD studies have been used to examine AMPs, a comprehensive compliling of all of these studies is beyond the scope of this work.

Various applications and web servers available for the prediction and design of AMPs are shown in Table 3.

## 7. Discussion

The Infectious Diseases Society of America (IDSA) identified a group of particularly problematic pathogens, termed ESKAPE pathogens (*E. faecium*, *S. aureus*, *K. pneumoniae*, *A. baumannii*, *P. aeruginosa*, *Enterobacter* spp.), requiring urgent effective treatments [596]. These pathogens are known to ‘escape’ the bactericidal effects of many antibiotics via multiple drug resistance mechanisms, rendering them almost totally resistant to existing antibiotics [597]. Consequently, interest in AMPs as an alternate therapy for infections caused by such resistant organisms has increased over the last two decades [598]. Here, we have summarised the sources, classification, and mechanism of actions of AMPs, as well as examining a variety of AMP databases and AMP development tools. A unique advantage of AMPs compared to traditional antibiotics is that they have multiple biological targets [599]. This is exemplified by LL-37, which acts on the bacterial cell membrane but also exhibits direct microbicidal, immune modulation, and antibiofilm activity [600,601]. It is therefore not surprising that AMPs are increasingly being examined as potential alternate therapy for infections caused by MDR pathogens [598]. However, of the thousands of AMPs discovered, the United States Food and Drug Administration (FDA) has only approved the glycopeptides (vancomycin, oritavancin, dalbavancin, and telavancin) and daptomycin for use against Gram-positive bacteria, the polymyxins (polymyxin B and colistin [polymyxin E]) for use against Gram-negative bacteria, and gramicidin for use against both Gram-type bacteria [602]. In many countries in Asia and Europe, teicoplanin (a glycopeptide) is also used clinically for the prophylaxis and treatment of serious infections caused by Gram-positive bacteria [603]. The nikkomycins and echinocandins are AMPs which are currently being investigated for the treatment of fungal infections caused by *Blastomyces dermatitidis*, *Aspergillus niger*, and *C. albicans* [506].

While AMPs have potential therapeutic benefits compared to existing antibiotics, they also come with certain limitations that hinder their development for use in the clinic. Natural AMPs typically have poor absorption, distribution, metabolism, and excretion (ADME) properties, as well as a short half-life and low permeability and solubility [604]. These properties have proven to be a major hindrance to the development of novel AMP treatments. Several studies have demonstrated that certain properties of AMPs, including ADME, cytotoxicity, and proteolytic stability, can be modulated by altering the peptide composition and post translational modifications [605,606,607]. Strategies to help turn peptides into potentially useful medicines have been comprehensively reviewed elsewhere [604]. Prediction tools utilizing machine learning algorithms with an accuracy, sensitivity, and specificity of ≥ 90% have accelerated peptide discovery as well as resistance-gene prediction in microbial genomes [608]. The in silico approach of MD simulations has provided a better understanding of structure–activity relationships (SARs), including mechanisms of action and the identification of important residues contributing to antibacterial activity [609]. Recent work by Zhu et al. elucidated polymyxin-dependent resistance using MD simulations with other omics approaches [610], while Jiang et al. used MD simulations to reveal the structure-interaction relationship of polymyxins with the lipid A-based outer membrane of *A. baumannii* [611]. When combined with experimental validation, MD simulation can elucidate the detailed mechanisms of action at the atomic level, making it especially useful for novel AMP development [552]. Recently, Chen et al. described a new simulation-guided rational design for the development of a small-pore forming AMP [612], whereas Kleandrova et al. described a new method for simultaneously predicting AMP antibacterial activity and cytotoxicity [613].

The conserved structures, specific targeted activities, ease of synthesis, and small size make AMPs promising therapeutic agents. Synthetic and chemical biology has great potential to develop AMPs with enhanced antimicrobial activity and reduced toxicity [614,615]. Improvements in MD simulation algorithms and machine learning strategies that will inevitably come with increasing computational power and high spec dedicated systems will greatly assist in the prediction and determination of AMP SARs. Similarly, improved molecular dynamics and deep learning algorithms can be developed to extract extensive features from already reported antimicrobial, non-antimicrobial, cytotoxic, and non-cytotoxic AMPs. The mining of halicin for resistant infections is a promising example of deep learning in the drug discovery and development pipline [616]. Similarly, the advent of alpha fold, which represents a significant breakthrough in computational biology, exemplifies the promise of deep learning approaches in AMPs discovery [540]. A network biology approach can also be adopted for peptide-induced pathways and peptide–protein and peptide–gene interactions, enhancing our understanding of AMP functioning. Finally, the development of robust tools that can simultaneously detect AMP activity, mode of action, cytotoxicity, and other adverse effects would be of great benefit. Nevertheless, wet laboratory assays for determining cytotoxicity as well as activity are still be required.

Overall, we systematically summarize the recent significant progress of AMP on origins, sequences, classifications, structures, and databases. The knowledge and insights would be valuable for drug discovery and treatment development to combat a variety of infectious diseases.

## Figures and Tables

**Table 1:** Classification of Plant AMPs.

Class	Description	Activity	References
Thionins	Cationic peptides of 45–48 amino acids containing 3–4 disulfide bridges	Antibacterial, Antifungal	[149,151]
Defensins	Catoinic peptides of 45 to 54 amino acids containing 4–5 disulfide bridges	Antibacterial, Antifungal	[152,153]
Hevein-like peptides	Basic peptides of 29–45 residues containing 3–5 disulfide bridges, rich in glycine and aromatic residues	Antifungal	[154,155]
Knottin-typepeptides	Peptides of ~30 residues, consisting of conserved cysteine residues and disulfide bridges	Antiviral, Antibacterial	[149,156]
α-Hairpinins	Rich in lysine/arginine, containing a helix-loop-helix secondary structure	Anti-HIV, Antibacterial, Antifungal	[149,157,158,159]
Lipid Transfer Proteins	Cationic peptides containing 70–90 residues that includes 8 cysteine residues	Antibacterial, Antifungal	[149,160,161]
Snakins	Catoinic small sized proteins characterized by 12 conserved cysteine residues	Antibacterial, Antifungal	[162,163]
Non-Cysteine Rich Peptides	Containing 0–1 cysteine residues and possessing high structural flexibility	Antibacterial, Antifungal	[149,164,165]

**Table 2:** Classification of Amphibians AMPs and their activities.

Class	Description	Activity	References
Bominin	Glycine-rich peptides with *C*-terminal amidated residues, isolated from skin secretions of *Bombina* genus	Antibacterial	[236,237]
Buforin	Cationic peptides rich in arginine and lysine, DNA-targeting, isolated from the stomach of *Bufo gargarizans*	Antibacterial, Antifungal	[238,239]
Cathelicidin	Amphibian cathelicidins possess homology with mammalian cathelicidins. Over 20 cathelicidins have been identified	Antibacterial	[240,241]
Dermaseptin	Cationic AMPs with an amphipathic structure of 24–34 residues. Dermaseptins are derived from the skin of the tree frog *Phyllomedusa sauvagii* and are involved in protein synthesis inhibition and induction of apoptosis	Antiviral, Antibacterial, Antifungal, Anticancer	[242,243,244]
Esculentin	Originally found in *Rana tigrina* (an edible frog). Characterized by *C*-terminal disulfide bond	Antibacterial, Antifungal	[245,246]
Fallaxin	A 25-residue AMP known as ocellatin-F1; isolated from *Leptodactylus fallax*	Antibacterial, Leishmanicidal	[247,248]
Maximin	Derived from toad related species, e.g., Chinese red belly toad (*Bombina maxima*)	Antibacterial	[249,250]
Magainin	Alpha-helical peptides isolated from the skin of *X. laevis*; interact with membrane phospholipids leading to disruption of the ionic gradient	Antibacterial, Antifungal, Antitumor	[251,252,253]
Plasticin	Dermaseptin-like peptides with 23–29 amino acids; active agasint bacteria via membrane disruption and pore formation	Antibacterial	[254,255]
Palustrin	Isolated from *Lithobates Palustris*. Characterized by the presence of disulfide bond and the rana box domain	Antibacterial	[256,257]
Phylloxin	Bacteriostatic AMPs; insolated from species including *Phyllobates bicolor.*	Antibacterial	[258]
Phyllospetin	*C*-terminal amidated peptides of 20 amino acids; isolated from South American tree frogs; inducing membrane permeabilization via a carpet-like action	Antibacterial, Antifungal, Antiparasitic, Anticancer	[259,260]
Psuedin	Isolated from the skin of *Psuedis paradoxa*; RNA targeting, ultimately stopping protein synthesis	Antibacterial, Antifungal	[261,262]
Ranateurin	Derived from the American bullfrog *Rana catesbeiana*	Antibacterial, Anticancer	[263]
Ranalexin	A *C*-terminal heptapeptide ringed 20 amino acids AMP having one disulfide bond	Antibacterial, Antifungal, Antiparasitic	[264,265]

**Table 3:** Tabulated representation of AMP databases/servers for prediction.

Server	Algorithm	Year	Description	URL	Ref
CyBase	ellipsoid and random walk algorithm	2008	database of cyclic protein sequences and structures	http://www.cybase.org.au/index.php (accessed on 22 October 2021)	[567]
AntiBP2	SVM	2010	Predict antibacterial peptides in protein sequences	www.imtech.res.in/raghava/antibp/ (accessed on 2 October 2020)	[568]
THIOBASE	-	2011	Database of thiopeptides	https://bioinfo-mml.sjtu.edu.cn/THIOBASE/index.php (accessed on 22 October 2021)	[569]
AvPred	SVM	2012	Predict antiviral peptides	http://crdd.osdd.net/servers/avppred/index.html (accessed on 2 October 2020)	[570]
ThioFinder	HMMs	2012	Identify thiopeptide antibiotic gene clusters in DNA sequences	https://bioinfo-mml.sjtu.edu.cn/ThioFinder/index.php (accessed on 22 October 2021)	[569]
ClassAMP	RF, SVM	2012	Predict AMP domains in protein sequences	http://www.bicnirrh.res.in/classamp/ (accessed on 2 October 2020)	[571]
Mutator 2.0	Mutator Algorithm	2012	Predict the effect of single or double amino acid substitutions on the therapeutic index (TI) of helical AMPs	http://split4.pmfst.hr/mutator/ (accessed on 2 October 2020)	[572]
DADP	-	2012	Database of bioactive peptides from anuran	http://split4.pmfst.hr/dadp/ (accessed on 22 October 2021)	[573]
YADAMP	DSC	2012	AMP database of and predict AMPs	http://yadamp.unisa.it/about.aspx (accessed on 22 October 2021)	[574]
CPPpred	N-to-1 NN	2013	Predict cell penetrating peptides	http://bioware.ucd.ie/~compass/biowareweb/Server_pages/cpppred.php (accessed on 2 October 2020)	[575]
HIPdb	PepStr algorithm	2013	Database of experimentally validated anti-HIV Peptides	http://crdd.osdd.net/servers/hipdb/ (accessed on 2 October 2020)	[576]
ADAM	HMM, SVM	2015	Predict AMPs	http://bioinformatics.cs.ntou.edu.tw/ADAM/tool.html (accessed on 2 October 2020)	[577]
DBAASP	New algorithm DBSCAN	2015	Predict AMPs	https://dbaasp.org/home (accessed on 2 October 2020)	[516]
BaAMPs	-	2015	Database of anti-biofilm AMPs	http://www.baamps.it/ (accessed on 22 October 2021)	[578]
CAMP_R3_	SVM, RF, ANN, DA	2016	Predict AMPs	http://www.camp3.bicnirrh.res.in/index.php (accessed on 2 October 2020)	[549]
APD3	Peptide Parameter Space	2016	Predict AMPs	http://aps.unmc.edu/AP/main.php (accessed on 2 October 2020)	[234]
dPABBs	SVM, WEKA	2016	Predict and design anti-biofilm peptides	http://ab-openlab.csir.res.in/abp/antibiofilm/ (accessed on 2 October 2020)	[579]
MBPDB	-	2017	Database of bioactive peptides derived from milk protein	http://mbpdb.nws.oregonstate.edu/ (accessed on 22 October 2021)	[580]
RiPPMiner	SVM, WEKA	2017	Decipher RiPPs from amino acid sequence of precursor polypeptide.	http://www.nii.ac.in/rippminer.html (accessed on 22 October 2021)	[581]
iAMPpred	SVM	2017	Predict AMPs	http://cabgrid.res.in:8080/amppred/ (accessed on 22 October 2021)	[582]
InverPep	CALCAMP/in-house algorithm	2017	Database of experimentally validated AMPs from invertebrates	https://ciencias.medellin.unal.edu.co/gruposdeinvestigacion/prospeccionydisenobiomoleculas/InverPep/public/home_en (accessed on 22 October 2021)	[583]
BAGEL4	HMM, Genomic context	2018	Predicts RiPPs and bacteriocins	http://bagel4.molgenrug.nl (accessed on 2 October 2020)	[584]
AMPscanner Vr.1	RF & MARS	2018	Predict AMPs	https://www.dveltri.com/ascan/v1/index.html (accessed on 2 October 2020)	[585]
AMPscanner Vr.2	DNN	2018	Predict AMPs	https://www.dveltri.com/ascan/v2/about.html (accessed on 2 October 2020)	[585]
dbAMP	RF/BLASTP	2019	Predict AMPs	http://140.138.77.240/~dbamp/index.php (accessed on 2 October 2020)	
ADAPT-ABLE	SR family generating, CF algorithm	2019	Data mining and AMP prediction	http://gec.u-picardie.fr/adaptable (accessed on 2 October 2020)	[515]
AMAP	SVM, XGBoost	2019	Predict biologically active peptides and AMPs.	http://amap.pythonanywhere.com/ (accessed on 2 October 2020)	[586]
AntiVPP1.0	RF	2019	Predict antiviral peptides.	https://github.com/bio-coding/AntiVPP (accessed on 2 October 2020)	[587]
Meta-iAVP	k-NN, rpart, glm, RF, XGB, SVM	2019	Predict antiviral peptides.	http://codes.bio/meta-iavp/ (accessed on 2 October 2020)	[588]
mACPpred	SVM	2019	Predict anticancer peptides.	www.thegleelab.org/mACPpred (accessed on 2 October 2020)	[589]
Deep-AmPEP30	CNN	2020	Predict AMPs ≤30 residues.	https://cbbio.cis.um.edu.mo/AxPEP (accessed on 2 October 2020)	[590]
AmpGram	RF	2020	Predict and design AMPs from proteomic data.	http://biongram.biotech.uni.wroc.pl/AmpGram/ (accessed on 2 October 2020)	[591]
IAMPE	NB, KNN, SVM, RF, and XGBoost	2020	Predict physicochemical and NMR features of peptides.	http://cbb1.ut.ac.ir/ (accessed on 2 October 2020)	[592]
CancerGram	n-grams and random forests	2020	Predict anticancer peptides.	http://biongram.biotech.uni.wroc.pl/CancerGram/ (accessed on 2 October 2020)	[593]
ACEP	DNN	2020	Predict AMPs	https://github.com/Fuhaoyi/ACEP (accessed on 2 October 2020)	[594]
AniAMPpred	SVM, DNN	2021	Identify the antimicrobial function of proteins	https://aniamppred.anvil.app/ (accessed on 22 October 2021)	[548]
DRAMP 3.0	-	2021	Data repository of antimicrobial peptides and predict AMPs	http://dramp.cpu-bioinfor.org/ (accessed on 22 October 2021)	[595]

ANN, artificial neural network; CNN, convolutional neural network; DA, discriminant analysis; DBSCAN, density-based clustering algorithm; DNN, deep neural network; FKNN, fuzzy K-nearest neighbor; glm, generalized linear model; HMM, hidden Markov model; MARS, Multivariate adaptive regression spline; NB, Naïve Bayes; RF, random forest; rpart, recursive partitioning and regression trees; SVM, support vector machine; WEKA, Waikato Environment for Knowledge Analysis; XGBoost, extreme gradient boosting.
